# Effects of *Passiflora incarnata* Leaf Polyphenol Extract on High-Fat-Diet-Induced Metabolic Syndrome in Mice and Associated Changes in the Gut–Liver–Adipose Axis

**DOI:** 10.3390/foods15173078

**Published:** 2026-08-30

**Authors:** Ying Ju, Liangliang Qi, Yongcai Huang, Ge Chen, Jie Gui, Xuzhou Liu, Liu Yang

**Affiliations:** 1 Guangxi Crop Genetic Improvement and Biotechnology Laboratory, Guangxi Academy of Agricultural Sciences, Nanning 530007, China; 13578935348@163.com (Y.J.); huangyongcai1981@126.com (Y.H.); cgprincess@gxaas.net (G.C.); guijie142429@163.com (J.G.); 2 Institute of Microbiology, Guangxi Academy of Agricultural Sciences, Nanning 530007, China; gx_macrofungi@126.com

**Keywords:** *Passiflora incarnata* leaf polyphenols, obesity, gut–liver–adipose axis, adipose thermogenesis

## Abstract

Metabolic syndrome, driven by obesity, poses a significant global health burden. *P. incarnata* leaf polyphenol extract (PP), a crude 70% ethanol extract, exhibits potential bioactive properties, yet its efficacy and underlying mechanisms against metabolic disorders remain unclear. This study investigated effects of this extract (PP) on high-fat diet (HFD)-induced metabolic syndrome in mice and explored possible involvement of the gut–liver–adipose axis. Thirty mice were divided into five groups: ND (normal diet), HFD, PC-HFD (HFD + orlistat), PP1 (HFD + 75 mg/kg/day PP), and PP2 (HFD + 150 mg/kg/day PP). After 8 weeks, high-dose PP (PP2) significantly attenuated body weight gain, and reduced food intake, hyperlipidemia, and systemic inflammation. In addition, PP intervention altered the gut microbiota composition and was associated with enrichment of beneficial genera and elevated fecal short-chain fatty acids (SCFAs). Hepatic transcriptomics showed that PP2 extensively reversed HFD-induced transcriptional suppression, while fecal metabolomics indicated restoration of key metabolite profiles. Furthermore, PP2 markedly upregulated thermogenic gene expression in adipose tissue. Collectively, these findings suggest that PP, particularly at the high dose, ameliorated HFD-induced obesity and related metabolic disturbances; these benefits were accompanied by decreased caloric intake, changes in gut microbiota composition, hepatic transcriptomic profiles, and adipose thermogenic gene expression.

## 1. Introduction

Obesity and its associated metabolic syndrome, driven by a chronic imbalance between energy intake and expenditure, have become major global public health challenges [1]. In this context, enhancing energy dissipation through the activation of adipose tissue thermogenesis has emerged as a promising strategy to counteract metabolic disorders. Mammalian adipose tissue serves not only as an energy storage depot but also as an active endocrine organ. While white adipose tissue (WAT) primarily stores energy, brown and beige adipose tissues dissipate energy via mitochondrial uncoupling protein 1 (UCP1)-mediated non-shivering thermogenesis. Consequently, inducing the “browning” of WAT to enhance thermogenesis represents a highly promising anti-obesity approach [2,3].

Plant polyphenols have garnered significant attention for their anti-obesity and insulin-sensitizing effects [4]. These compounds exert multi-target regulation on energy metabolism, primarily converging on the AMPK/PGC-1α/UCP1 signaling axis to promote mitochondrial biogenesis and thermogenesis [5]. Notably, emerging evidence highlights that the metabolic benefits of polyphenols are highly dependent on the gut microbiota. The “gut microbiota–metabolite” axis plays a pivotal role in regulating distal adipose thermogenesis. For instance, resveratrol and blueberry extract require an intact gut microbiota to exert their anti-obesity effects, which are often abolished in antibiotic-treated models [6,7]. Mechanistically, polyphenols enrich beneficial bacteria (e.g., *Akkermansia muciniphila*) and elevate microbial metabolites such as short-chain fatty acids (SCFAs) and secondary bile acids. These metabolites, particularly butyrate and lithocholic acid, act as signaling molecules to activate the TGR5/cAMP/PKA and AMPK pathways, thereby upregulating UCP1 expression and driving adipose tissue browning [8,9,10].

The *Passifloraceae* family, comprising over 600 species, is renowned for its diverse chemical composition and pharmacological potential [11,12]. *Passiflora incarnata* leaves are particularly rich in bioactive polyphenols (133.7 mg GAE/g), demonstrating potent antioxidant capacity through strong reducing power and free radical scavenging [13]. Previous studies have established that dried leaf extracts of *P. incarnata* effectively ameliorate high-fat diet (HFD)-induced dyslipidemia, reducing total cholesterol (TC) and triglycerides (TG), while elevating high-density lipoprotein (HDL) levels. Furthermore, these extracts mitigate systemic inflammation and hepatic oxidative stress, as indicated by reductions in C-reactive protein and alkaline phosphatase, and prevent left ventricular hypertrophy [14].

In HFD-fed C57BL/6J mice, a *P. incarnata* extract (60% ethanol, C-glycosyl flavone-rich) reduced body weight, serum lipids, hepatic fat, and adipocyte size, with vicenin-2 as a key anti-lipogenic agent [15]. Similarly, green tea polyphenols (0.05–0.2% diet) dose-dependently lowered lipids, glucose, and insulin; increased fecal SCFAs; and reversed gut dysbiosis in HFA mice [16]. Grape seed proanthocyanidins (200 mg/kg/day) reduced weight, improved insulin sensitivity, upregulated thermogenic markers, and normalized gut microbiota [17]. Together, these findings support targeting the gut–liver–adipose axis with polyphenol-rich extracts for metabolic syndrome.

Therefore, we employed an integrated multi-omics approach, combining 16S rRNA sequencing, fecal metabolomics, hepatic transcriptomics, and targeted gene expression analysis, to systematically evaluate the effects of *Passiflora incarnata* leaf polyphenol extract (PP) supplementation on HFD-induced metabolic syndrome and to explore possible associated changes in gut microbiota composition, metabolic profiles, and adipose thermogenic gene expression.

## 2. Materials and Methods

### 2.1. Isolation and Characterization of Polyphenol Extract from P. incarnata Leaves

*P. incarnata* leaves were obtained from plants cultivated by our research group. The plant species was authenticated by Prof. Liu Yang (Guangxi Academy of Agricultural Sciences). Polyphenol extract from the leaves of *P. incarnata* was extracted using an ultrasonic-assisted method [18]. Briefly, dried leaf powder was mixed with 70% ethanol as the solvent, at a solid-to-liquid ratio of 25:1 (mL/g). The mixture was then subjected to ultrasonic extraction at 350 W and 60 °C for 30 min. Following extraction, the solution was vacuum-filtered. Ethanol was removed from the filtrate by rotary evaporation under reduced pressure at a temperature below 50 °C. The resulting concentrate was subsequently freeze-dried to obtain a crude polyphenol extract powder. Extraction yield was calculated as (mass of freeze-dried extract/mass of dried leaf powder) × 100%. Total phenolic content (TPC) was determined by the Folin–Ciocalteu colorimetric assay. Under alkaline conditions, phenolic hydroxyl groups reduce the Folin–Ciocalteu reagent to form a blue complex, and the absorbance was measured at 765 nm. Gallic acid was used to establish the calibration curve, and the results were expressed as milligrams of gallic acid equivalents per gram of crude extract powder (mg GAE/g extract).

The polyphenol composition was analyzed using a Thermo Fisher Scientific (Waltham, MA, USA) UHPLC-Exploris 240 ultra-high-performance liquid chromatography system coupled with Fourier-transform mass spectrometry (UHPLC-FT-MS). Chromatographic separation was performed on an ACQUITY UPLC HSS T3 (Milford, MA, USA) column (100 mm × 2.1 mm, 1.8 µm) maintained at 40 °C, with an injection volume of 3 µL. Mobile phase A consisted of 95% water/5% acetonitrile containing 0.1% formic acid, and mobile phase B consisted of 47.5% acetonitrile/47.5% isopropanol/5% water containing 0.1% formic acid. Mass spectrometry analysis was conducted using an electrospray ionization source, with data acquired in both positive and negative ion modes. The full scan range was set at *m*/*z* 70–1050, with a resolution of 60,000 for full MS and 15,000 for MS/MS. The main mass spectrometry parameters were set as follows: sheath gas flow rate at 60 arb; auxiliary gas flow rate at 20 arb; ion transfer tube temperature at 320 °C; vaporizer temperature at 350 °C; spray voltage at 3400 V for positive mode and −3000 V for negative mode; and normalized collision energies at 20%, 40%, and 60%.

Compound annotation was performed based on multiple orthogonal evidence: (1) accurate monoisotopic mass (mass tolerance ≤ 5 ppm); (2) matching of experimental MS/MS fragmentation patterns with reference spectra from public databases (mzCloud, ChemSpider, and HMDB); and (3) retention-time consistency with authentic standards analyzed under identical UHPLC conditions and with database entries reporting retention time for comparable reversed-phase systems. Where available, authentic reference standards were used for definitive identification via simultaneous matching of retention time, exact mass, and MS/MS fragments. Compounds without access to commercial standards were tentatively annotated according to the above mass spectrometric and chromatographic evidence. Authentic standards used for quantification (gallic acid) and qualitative retention-time/MS^2^ confirmation (chlorogenic acid, vitexin, isovitexin, rutin, and quercetin-3-O-glucoside) were analyzed in-house.

### 2.2. Prediction of Bioactive Compounds

The polyphenolic composition of PP was determined by liquid chromatography for subsequent network pharmacology analysis. The CAS numbers of the detected components were cross-referenced with the Traditional Chinese Medicine Systems Pharmacology Database (TCMSP; https://www.tcmsp-e.com/index.php (accessed on 20 August 2025)). A compound was considered biologically active if it met both of the following criteria: oral bioavailability (OB) ≥ 20% and drug-likeness (DL) ≥ 0.1. For compounds not found in the TCMSP database, further analysis was performed using the Swiss ADME platform (http://www.swissadme.ch/index.php (accessed on 20 August 2025)). In this case, a compound was deemed biologically active if it demonstrated high gastrointestinal absorption (GI absorption) and satisfied at least two drug-likeness criteria (indicated as “yes”).

### 2.3. Intersection Analysis of Targets Between PP and Lipid Metabolism

Following the acquisition of bioactive compounds, systematic target prediction for PP active ingredients was conducted using the Swiss Target Prediction database (https://www.swisstargetprediction.ch/ (accessed on 20 August 2025)). The canonical SMILES structures of each active component were obtained from the PubChem database (https://pubchem.ncbi.nlm.nih.gov/) and subsequently submitted to the Swiss Target Prediction online platform, with the species parameter specified as “Homo sapiens”. To ensure comprehensive target coverage, experimentally validated known targets were additionally retrieved using the “Related Targets” function in the TCMSP database. All predicted targets were then standardized through the UniProt database (https://www.uniprot.org/), where protein designations were converted to official gene symbols, with subsequent removal of targets lacking corresponding gene information or presenting as duplicates. The final reliable target dataset for PP active ingredients was established by integrating prediction results from Swiss Target Prediction with documented target information from the TCMSP database.

To systematically identify potential targets associated with lipid metabolism disorders, we integrated three authoritative disease databases (OMIM, GeneCards, and DisGeNET) using standardized screening criteria. The specific methodology was as follows: First, we searched all three databases using the keywords “hyperlipidemia” and “lipid metabolism”. From GeneCards, we selected disease-related genes with a relevance score ≥ 10; from DisGeNET, we filtered disease genes with Score_gda ≥ 0.1; and from OMIM, we included all relevant genes. After merging the gene datasets from these three sources, we removed duplicates using bioinformatics methods to establish a comprehensive lipid metabolism disease target library. Subsequently, both the active targets of PP and the lipid metabolism disease targets were imported into the Venny online tool (https://bioinfogp.cnb.csic.es/tools/venny/ (accessed on 20 August 2025)) for intersection analysis. The overlapping targets identified through this approach were considered as potential therapeutic targets through which PP may exert its anti-obesity effects. This integrated screening strategy ensures both reliability and representativeness of the target data for subsequent mechanistic investigations.

### 2.4. Core Target Gene Screening and Enrichment Analysis

The common interaction targets were submitted to the STRING database (https://string-db.org/), with Homo sapiens designated as the target species for protein–protein interaction (PPI) network analysis. The resulting data were subsequently imported into Cytoscape 3.10.1 software (https://cytoscape.org/) for core target identification. Core targets were selected based on three key topological parameters-degree centrality, betweenness centrality, and closeness centrality-with the threshold criteria set at values exceeding the median for all three metrics. Kyoto Encyclopedia of Genes and Genomes (KEGG) pathway enrichment analyse of these core targets was then performed using the Metascape platform (http://metascape.org/gp/). To elucidate the mechanistic basis of PP active components in obesity amelioration, a multidimensional “active component-core target-signaling pathway” network was constructed in Cytoscape 3.10.1, which systematically integrated three fundamental elements: the bioactive constituents of PP, their key molecular targets, and the associated regulatory signaling pathways.

To address potential species differences, we verified the homology of core targets between Homo sapiens and Mus musculus using the NCBI HomoloGene database. All identified targets were confirmed to possess unambiguous one-to-one orthologs in mice, supporting the biological applicability of the human-based network predictions to our murine experimental system.

### 2.5. Animals and Treatment

Thirty male C57BL/6J mice, aged 6 weeks, with an initial body weight of 19.42 ± 0.79 g, were obtained from Hunan Slake Jingda Experimental Animal Co., Ltd (Changsha, China). The mice were maintained under controlled environmental conditions, including a 12 h light/dark cycle, relative humidity of 40–60%, and a temperature range of 20–22 °C. They were provided with ad libitum access to food and water.

Following a one-week acclimatization period, the mice were randomly assigned to one of five experimental groups using a computer-generated random number table, with 6 mice per group. The five groups were as follows: ND (normal diet; SY10001F maintenance feed, purchased from Shanghai Shuyu Biotechnology Co., Ltd. (Shanghai, China), with 21% kcal from protein, 11% kcal from fat, and 68% kcal from carbohydrate; fat sources included soybean oil, fish meal, and chicken meal); HFD (high-fat diet; SYHF60-1, purchased from Shanghai Shuyu Biotechnology Co., Ltd., with 19.09% kcal from protein, 58.79% kcal from fat, and 22.12% kcal from carbohydrate; fat sources were primarily lard and whole milk powder); PC-HFD (HFD supplemented with orlistat at 20 mg/kg/day); PP1 (HFD supplemented with PP at 75 mg/kg/day); and PP2 (HFD supplemented with PP at 150 mg/kg/day). The dosage range was selected based on the regimens reported in a previous study [19], which administrated *P. incarnata* leaf extract at 75–150 mg/kg/day in mice.

Orlistat and PP were administered daily via oral gavage, while the ND and HFD groups received an equivalent volume of ultrapure water. For PP administration, the crude polyphenol extract powder was suspended in ultrapure water at concentrations of 7.5 mg/mL (for PP1) and 15 mg/mL (for PP2) based on a gavage volume of 0.1 mL/10 g body weight. The suspension was vortexed vigorously for 30 s and sonicated for 1 min at room temperature to achieve a homogeneous suspension, and it was freshly prepared each day. Immediately before each gavage, the suspension was re-vortexed to prevent sedimentation. To ensure blinding, the investigator responsible for gavage administration was not involved in subsequent outcome measurements. Additionally, all experimental groups received the same volume (0.1 mL/10 g) of either ultrapure water or test suspensions to maintain uniformity of handling. Throughout the study, no gavage needle blockage was observed. Body weight was monitored on a weekly basis throughout the study, while feed intake was recorded daily by weighing the remaining food pellets and calculating the difference between the amount provided and the amount remaining.

After 8 weeks of treatment, mice (*n* = 6 per group; 30 mice total) were fasted for 12 h and subsequently euthanized using sodium pentobarbital (50 mg/kg). Blood samples were collected via enucleation, and fasting blood glucose levels were determined. To maintain blinding during the analytical phase, all tissue samples were coded with random numbers before weighing and biochemical measurements, and the codes were broken only after the complete dataset was acquired. The weights of the whole body, epididymal fat pads, and liver were recorded. Tissue samples, including liver, feces, serum, and adipose tissue, were collected for further analysis. Organs were carefully dissected and weighed, and the organ index (%) was calculated using the following formula: (Organ Weight (g)/Body Weight (g)) × 100%.

### 2.6. Serum Biochemistry, Inflammatory Cytokines, and Hepatic Antioxidant Enzymes

Lipid parameters, including TG, TC, low-density lipoprotein cholesterol (LDL-C), and high-density lipoprotein cholesterol (HDL-C), were quantified in each group using commercially available kits from Jiancheng Institute (Nanjing, China) and a biochemical analyzer (Model 7170, Tokyo, Japan). Blood glucose concentrations were determined using a glucose assay kit purchased from Shanghai Rongsheng Biotech (Shanghai, China). Serum concentrations of interleukin-6 (IL-6), interleukin-1 β (IL-1β), and tumor necrosis factor-α (TNF-α) were determined using commercial ELISA kits (MLBio, Shanghai, China), following the manufacturer’s instructions.

Liver homogenates were prepared as per the kit instructions for the assessment of antioxidant-related parameters. The activities of glutathione peroxidase (GSH-Px), superoxide dismutase (SOD), catalase (CAT), and malondialdehyde (MDA) were determined colorimetrically with commercially available kits (Jiancheng Institute, Nanjing, China), strictly adhering to the manufacturer’s instructions.

### 2.7. Analysis of Short-Chain Fatty Acids (SCFAs) and Histological Assessment

For SCFAs analysis, fecal samples were processed according to established protocols for fecal SCFA extraction [20]. Briefly, approximately 20 mg of feces was homogenized in 1 mL of 0.5% (*v*/*v*) phosphoric acid solution using a ball mill (30 Hz, 1 min), vortexed for 10 min, ultrasonicated for 5 min at 4 °C, and centrifuged at 12,000 rpm for 10 min at 4 °C. A 100 μL aliquot of the supernatant was transferred to a new tube and extracted with 500 μL of methyl tert-butyl ether (MTBE) containing an internal standard. After vortexing (3 min), ultrasonication (5 min at 4 °C), and centrifugation (12,000 rpm, 10 min, 4 °C), 200 μL of the organic layer was collected for analysis. SCFA concentrations were determined using gas chromatography–tandem mass spectrometry (GC–MS/MS) on an Agilent 8890-7000D system (Agilent Technologies, Santa Clara, CA, USA) equipped with a DB-FFAP capillary column (30 m × 0.25 mm i.d. × 0.25 μm film thickness; J&W Scientific, Folsom, CA, USA). Helium was used as the carrier gas at a flow rate of 1.2 mL/min. The injection volume was 1 μL with a split ratio of 5:1. The oven-temperature program was held at 50 °C for 1 min, raised to 220 °C at 18 °C/min, and held for 5 min. The injector-inlet and transfer-line temperatures were 250 °C and 230 °C, respectively. All samples were analyzed in multiple reaction monitoring (MRM) mode. A series of authentic standards—acetic acid (CNW), propionic acid (ANPEL), isobutyric acid (Dr. Ehrenstorfer), butyric acid (Sigma-Aldrich, St. Louis, MO, USA), 2-methylbutyric acid (Dr. Ehrenstorfer), isovaleric acid (Dr. Ehrenstorfer), valeric acid (CNW), 4-methylvaleric acid (TCI), caproic acid (Rhawn), and 5-methylhexanoic acid (Bepure, Xiamen, China)—all of chromatographic grade, were prepared in MTBE and stored at −20 °C. Calibration curves were constructed over a concentration range of 0.005–20 μg/mL (13 concentration levels) using internal-standard normalization. The linear regression coefficients (R^2^) exceeded 0.99 for all target SCFAs. The lower limit of quantification (LLOQ) was 0.005 μg/mL for each analyte.

For histological evaluation, epididymal fat tissues were fixed in 4% paraformaldehyde at room temperature and subsequently dehydrated in a graded ethanol series, cleared in xylene, and embedded in paraffin. For liver tissues designated for Oil Red O staining, samples were embedded in OCT compound. Liver frozen sections (4 µm) were cut using a cryostat (CRYOSTAR NX50, Thermo Fisher Scientific, Waltham, MA, USA); epididymal fat sections (4 µm) were cut using a microtome (RM2016, Leica, Shanghai, China). For H&E staining, eWAT sections were stained with hematoxylin and eosin using a commercial HE kit (G1003, Servicebio, Wuhan, China). For Oil Red O staining, liver frozen sections were fixed in fixative (G1101, Servicebio) for 15 min; stained with Oil Red O solution (G1015, Servicebio) for 8–10 min in the dark; differentiated in 60% isopropanol (Sinopharm, Beijing, China) sequentially for 3 s and 5 s; and counterstained with hematoxylin (G1004, Servicebio), followed by differentiation (G1039, Servicebio) and bluing (G1040, Servicebio).

Images of Oil Red O-stained liver sections were captured using a Nikon Eclipse Ci microscope, and H&E-stained eWAT sections using a Nikon Eclipse E100 microscope (Nikon Corporation, Tokyo, Japan), both equipped with NIKON DS-U3 digital cameras (Nikon Corporation, Tokyo, Japan). For quantitative analysis, at least five randomly selected non-overlapping fields per sample were captured at 200× magnification. The cross-sectional area (µm^2^) of epididymal adipocytes was measured using ImageJ software (version 1.54i, National Institutes of Health, USA). For liver sections, the percentage of Oil Red O-positive area (lipid area/total area) was quantified using ImageJ.

### 2.8. 16S rRNA Gene Sequencing

Microbial DNA was extracted from intestinal chyme of 30 samples (5 experimental groups; 6 biological replicates per group) using the EZNATM Soil DNA Kit (D5625-02, Omega Bio-Tek Inc., Norcross, GA, USA), following the supplier’s guidelines. The V3–V4 segments of the bacterial 16S rRNA gene were amplified through a two-step PCR method [21]. The forward primer 338F (5′-ACTCCTRCGGGAGGCAGCAG-3′) and reverse primer 806R (5′-GGACTACCVGGGTATCTAAT-3′) were utilized, incorporating 8 bp barcodes to enable sample multiplexing. The thermal cycling protocol consisted of an initial denaturation step at 95 °C for 3 min, followed by 40 cycles of denaturation at 95 °C for 30 s, annealing at 55 °C for 30 s, and elongation at 72 °C for 45 s, with a final extension at 72 °C for 10 min. The resulting PCR amplicons were purified from a 2% agarose gel using the AxyPrep DNA Gel Extraction Kit (Axygen Biosciences, Union City, CA, USA) and sequenced on the Illumina MiSeq PE300 platform (Illumina, Inc., San Diego, CA, USA). Raw reads were quality-filtered using fastp (v0.22.0) to remove adapter sequences, reads containing N bases, reads with >40% low-quality bases (quality score < 15), and reads shorter than 150 bp after trimming. Chimeric sequences were identified and removed using vsearch (v2.22.1) against the SILVA reference database. Amplicon sequence variants (ASVs) were generated using the Deblur algorithm (v1.1.1) via QIIME 2, and taxonomic assignment was performed against the SILVA 138.1 database. After quality filtering, each sample yielded approximately 45,000–144,000 effective tags (mean length: 411–425 bp; Q20 > 97%, Q30 > 92%), with effective bases ranging from 19 to 59 million nucleotides per sample.

For downstream statistical analyses, the ASV table was rarefied to the minimum sequencing depth to normalize sampling effort across samples. Alpha diversity was estimated using multiple indices, including observed ASVs, Chao1, ACE, Shannon, Simpson, Goods_coverage, and PD_whole_tree. Beta diversity was evaluated based on both weighted and unweighted UniFrac distances, and community structural differences were visualized using Principal Coordinate Analysis (PCoA), Principal Component Analysis (PCA), and Non-Metric Multi-Dimensional Scaling (NMDS). Differentially abundant taxa between groups were identified using Linear Discriminant Analysis Effect Size (LEfSe) with a logarithmic LDA score threshold of >4, wherein the factorial Kruskal–Wallis test was first applied to detect significant features, followed by pairwise Wilcoxon tests to assess biological consistency.

### 2.9. Untargeted Cecal Metabolomic Analysis

Six biological replicates per group (*n* = 6) were analyzed. For metabolomic analysis, cecal digesta samples (approximately 20 mg) were extracted with 400 μL of 70% methanol in water (*v*/*v*) containing L-2-chlorophenylalanine (0.02 mg/mL) as an internal standard. The mixture was vortexed for 3 min, sonicated in an ice-water bath for 10 min, vortexed again for 1 min, and incubated at −20 °C for 30 min. After centrifugation at 12,000 rpm for 10 min at 4 °C, 300 μL of the supernatant was transferred to a new tube and centrifuged again at 12,000 rpm for 3 min at 4 °C; finally, 200 μL of the supernatant was collected for LC-MS analysis. Quality control (QC) samples were prepared by pooling equal volumes of all individual sample extracts and were injected every 10 analytical runs to monitor system stability and reproducibility. The coefficient of variation (CV) was <30% for >75% of the detected metabolites, and QC sample Pearson correlation coefficients exceeded 0.99.

LC-MS analysis was performed using a Vanquish UHPLC system coupled to a Q Exactive HF-X mass spectrometer (Thermo Fisher Scientific, Waltham, MA, USA). Chromatographic separation was achieved on a Waters ACQUITY Premier HSS T3 column (Waters Corporation, Milford, MA, USA; 1.8 μm, 2.1 mm × 100 mm) maintained at 40 °C, with mobile phase A consisting of 0.1% formic acid in water and mobile phase B of 0.1% formic acid in acetonitrile. The gradient program was as follows: 0–2 min, 5%→20% B; 2–5 min, 20%→60% B; 5–6 min, 60%→99% B; 6–7.5 min, held at 99% B; 7.5–7.6 min, returned to 5% B; and 7.6–10 min, held at 5% B. The flow rate was 0.4 mL/min, the column temperature was 40 °C, and the injection volume was 4 μL. Data were acquired in both positive and negative electrospray ionization (ESI^+^ and ESI^−^) modes. Key MS parameters for ESI^+^ were as follows: spray voltage, 3.5 kV; sheath gas, 30 Arb; auxiliary gas, 5 Arb; ion transfer tube temperature, 320 °C; vaporizer temperature, 300 °C; full scan range, *m*/*z* 75–1000; resolution, 35,000; and AGC target, 1 × 10^6^. ESI^−^ parameters were identical, except for a spray voltage of 3.2 kV.

Raw data were processed using XCMS (version 4.9) for peak detection, alignment, and retention-time correction. Peak area data were normalized by sum normalization prior to statistical analysis. Metabolite identification was performed at MSI Level 2 (putatively annotated compounds) based on accurate mass (<5 ppm), MS/MS spectral matching against public databases (HMDB, Metlin), and an in-house library, as well as the metDNA algorithm, with a comprehensive identification score ≥ 0.5. Statistical evaluations, including principal component analysis (PCA) and orthogonal partial-least-squares discriminant analysis (OPLS-DA), were performed using the R package MetaboAnalystR (version 4. 0). The OPLS-DA model was validated by R^2^X, R^2^Y, and Q^2^ values, with Q^2^ >0.5 considered acceptable, and a 200-fold permutation test (*p* < 0.05 indicating a valid model). Differential metabolites were selected using a VIP threshold >1 and a *p*-value < 0.05 (Student’s *t*-test for two-group comparisons or ANOVA for multi-group comparisons), without additional correction for multiple comparisons. Pathway analysis was performed using the KEGG database, and pathway enrichment was assessed via Fisher’s exact test implemented in the scipy.stats package in Python (version 3.12.0) [22].

### 2.10. Liver Transcriptome Analysis

Total RNA was extracted from liver tissues (*n* = 6 biological replicates per group) using a commercial kit (Sangon Biotech, Shanghai, China). RNA quality was assessed using a Qubit 4.0 fluorometer for concentration measurement and a Qsep400 analyzer for RNA integrity evaluation, ensuring high-quality RNA was used for subsequent library construction. mRNA libraries were constructed through Oligo(dT) magnetic-bead enrichment of polyadenylated mRNA, followed by fragmentation, random-primed first-strand cDNA synthesis, second-strand cDNA synthesis, end-repair, A-tailing, adapter ligation, size selection, and PCR amplification.

The libraries were sequenced on the Illumina platform (paired-end, 150-bp read length). Raw reads were quality-filtered using fastp to remove adapter sequences, reads with >10% N bases, and reads with >50% low-quality bases (Q ≤ 20). After filtering, each sample yielded approximately 44–58 million clean reads, generating at least 6 Gb of clean data per sample with a Q30 percentage ≥ 94%. Clean reads were mapped to the Mus musculus reference genome (Ensembl release GRCm39.109) using HISAT2 (version 2.2.1). Gene expression levels were quantified using featureCounts and normalized as FPKM (fragments per kilobase of transcript per million mapped reads) [23]. Gene-level read counts were quantified using featureCounts. DESeq2 was applied to the raw count matrix for differential expression analysis, with *p*-values adjusted using the Benjamini–Hochberg method; genes with |log_2_Fold Change| ≥ 1 and FDR < 0.05 were considered significantly differentially expressed. FPKM (fragments per kilobase of transcript per million mapped reads) values were calculated for visualization of expression distributions but were not used as input for differential testing. Functional enrichment analysis for KEGG pathways was performed using a hypergeometric test.

### 2.11. Quantitative Real-Time PCR (qRT-PCR) Analysis

The dorsal adipose tissue RNA was isolated using TRIzol reagent (Invitrogen, Thermo Fisher Scientific, Carlsbad, CA, USA), and its concentration was measured using a spectrophotometer (ND-1000, Thermo Fisher Scientific, Waltham, MA, USA). The total RNA was then converted into cDNA using Superscript II reverse transcriptase (Invitrogen, USA) for qRT-PCR experiments. qRT-PCR was performed using SYBR Green Master Mix under the following cycling conditions: 95 °C for 3 min, followed by 40 cycles of 95 °C for 15 s and 60 °C for 30 s, with a final melting curve analysis (65–95 °C) to confirm single-product amplification. Post-run melting curve analysis revealed single-peak amplification for all primer pairs, indicating target-specific amplification and the absence of primer dimers. Each cDNA sample was run in triplicate (technical replicates), and 6 biological replicates (mice) were included per group. Gene expression levels were analyzed using the 2^−ΔΔCT^ method, with GAPDH as the reference gene for normalization [24]. No significant difference in GAPDH CT values was observed across experimental groups (one-way ANOVA, *p* > 0.05), confirming its suitability as an internal control. The primers used for qRT-PCR were designed and produced by Bioengineering Co., Ltd. (Shanghai, China), and their sequences are detailed in Table 1.

### 2.12. Statistical Analysis

The assumptions of normality and homogeneity of variance were assessed using the Shapiro–Wilk test and Levene’s test, respectively. For longitudinal body-weight data collected weekly over the 8-week intervention period, a repeated-measures ANOVA was performed with experimental group as the between-subject factor and time (week) as the within-subject factor; when the sphericity assumption was violated, the Greenhouse–Geisser correction was applied. For endpoint data (serum biochemical parameters, inflammatory cytokines, hepatic transcriptomics, fecal metabolomics, and adipose thermogenic gene expression), statistical analysis was performed using one-way ANOVA with the experimental group as the fixed factor, and post hoc comparisons between groups were conducted using Tukey’s honestly significant difference (HSD) test in SAS 9.2 for multiple-comparison correction. Results were considered statistically significant at *p* < 0.05, while values ranging from 0.05 to 0.10 were interpreted as indicating a tendency toward significance. All data met the assumptions of ANOVA; therefore, no non-parametric alternatives were applied.

## 3. Results and Discussion

### 3.1. LC-MS Analysis and Bioactive Prediction of PP

The ultrasonic-assisted extraction of *P. incarnata* leaves afforded a crude polyphenol extract powder with an average extraction yield of 12.1 ± 0.4% (*w*/*w*, *n* = 3, relative to dried-leaf weight). The total phenolic content of the crude extract powder was determined to be 81.7 mg GAE/g extract by the Folin–Ciocalteu colorimetric assay. Based on the extraction yield, the total phenolic content of the dried *P. incarnata* leaves was calculated to be 9.89 mg GAE/g DW. To comprehensively characterize its phytochemical composition, a non-targeted UPLC-MS analysis was performed, leading to the tentative identification of 265 compounds, which are systematically cataloged in Table S1. Based on the HMDB superclass classification, flavonoids (121 compounds), phenols (51 compounds), and isoflavonoids (21 compounds) represented the most abundant chemical classes.

To pinpoint the predominant bioactive constituents, we further assessed their relative abundance by comparing individual peak areas. Notably, five compounds dominated the polyphenolic profile, together accounting for a substantial portion of the total peak area based on their exceptionally high intensities. These compounds were 5-hydroxyconiferyl alcohol, 6″-caffeoylhyperin, 3,4-dihydroxyhydrocinnamic acid, hydroquinone, and 3-hydroxyflavone. Their high relative abundance suggests that they are major components of the extract, though their specific contributions to bioactivity require further targeted investigation. The comprehensive chromatographic parameters (retention time, *m*/*z*, and adducts) and MS fragmentation scores are provided in Table S1, offering a chemical basis for correlating specific polyphenolic components with the observed metabolic benefits.

The pharmacokinetic properties of the screened compounds were analyzed using the TCMSP and Swiss ADME platforms. The analysis results showed that 40 compounds met the criteria of oral bioavailability (OB) ≥ 20% and drug-likeness (DL) ≥ 0.10, while another 30 compounds satisfied the requirements for gastrointestinal absorption (GI absorption) and drug-likeness. Based on these results, a total of 70 potentially active PP components were screened in this study.

In the target identification stage, the Related targets function of the TCMSP database was used to obtain the target information of these 70 active components. Subsequently, the Uniprot database (http://www.uniprot.org/) was employed to standardize protein names (converting them to standard gene nomenclature). After data cleaning (removing targets that could not be matched to gene symbols) and deduplication, it was ultimately determined that the PP active components acted on 644 unique gene targets.

### 3.2. Target Intersection Analysis of PP and Lipid Metabolism

**Figure 1:**
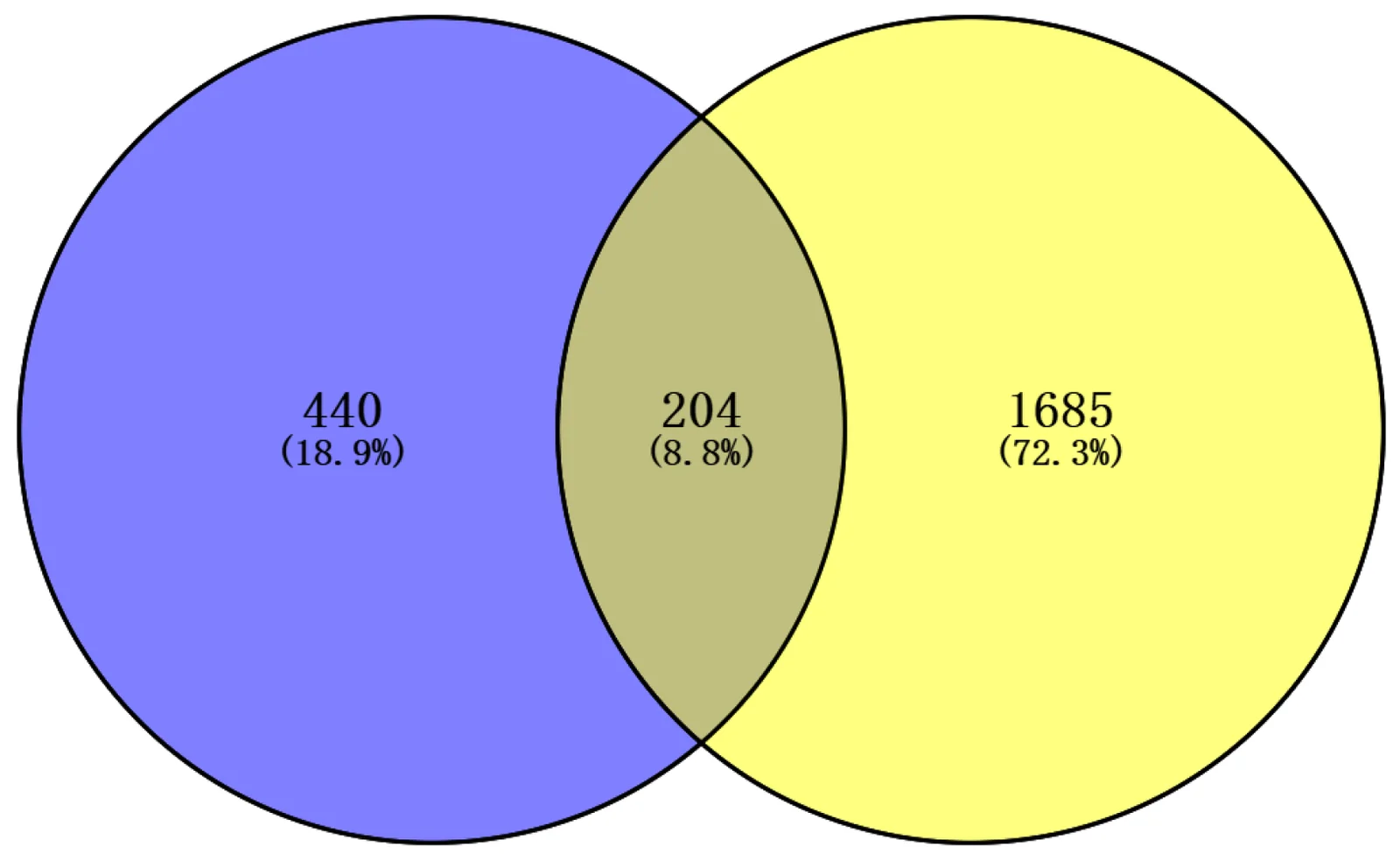
Intersection of targets of PP (**left**) and lipid metabolism (**right**).

By integrating the prediction results from both the Swiss Target Prediction and TCMSP databases, a total of 644 potential targets associated with the active ingredients of PP were identified. Meanwhile, lipid metabolism disorder-related targets were systematically retrieved from three major disease databases—OMIM, GeneCards, and DisGeNET. After standardization and removal of duplicates, 1889 targets related to lipid metabolism disorders were obtained. Intersection analysis of these two target sets was performed using the Venny online platform, revealing 204 common targets (Figure 1). These overlapping targets provide a computational basis for hypothesizing that PP active ingredients may influence lipid metabolic processes.

### 3.3. Core Targets and KEGG Enrichment Analysis

Network pharmacology was employed here as an exploratory tool for hypothesis generation, acknowledging its inherent limitations in predicting direct molecular interactions and its reliance on existing databases. Despite these constraints, this approach provided a rational foundation for identifying candidate targets and pathways that warrant further experimental validation. Employing the STRING database to construct a protein–protein interaction (PPI) network, topological analysis was subsequently performed using Cytoscape 3.10.1 software. Based on three key network topological parameters—degree centrality, betweenness centrality, and closeness centrality—a screening threshold was set to include only nodes exceeding the median value for all three metrics. Ultimately, 24 core targets were identified.

**Figure 2:**
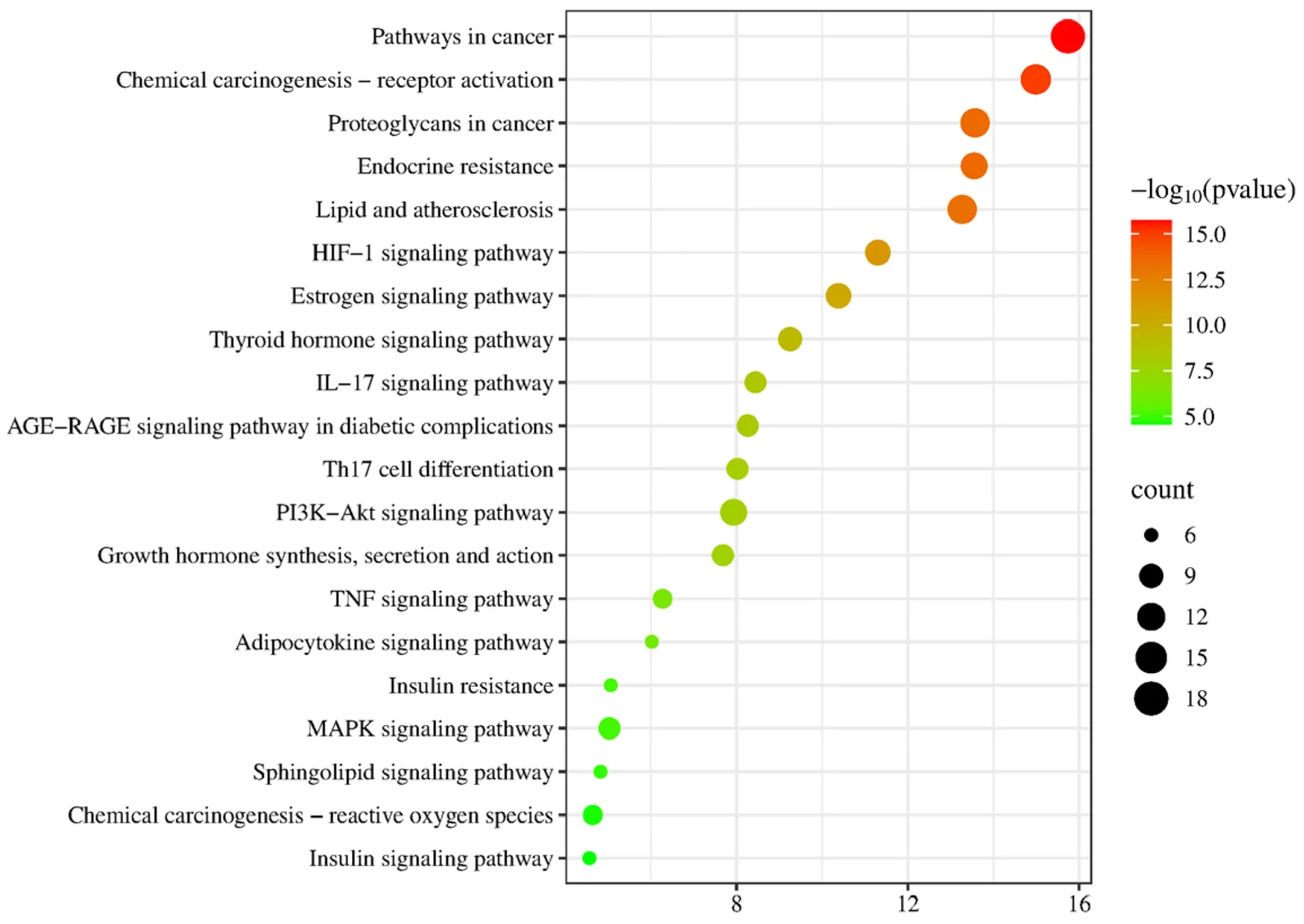
Top 20 enriched KEGG pathways for PP–lipid metabolism targets.

To further elucidate the potential biological functions of these targets, KEGG pathway enrichment analysis was performed on the core targets using the DAVID database. The top 20 pathways were selected based on significance levels (−log_10_ *p*-value) and visualized in a bubble chart (Figure 2). These pathways are predominantly involved in lipid metabolism (e.g., lipid and atherosclerosis), insulin signal transduction and resistance, adipocytokine signaling, inflammatory responses (e.g., TNF, IL-17, and AGE-RAGE signaling pathways), and endocrine hormone regulation (e.g., thyroid hormone and estrogen signaling pathways). These results suggest that PP may potentially exert its effects on lipid metabolism disorders, inflammatory stress, and metabolic homeostasis by modulating these pathways.

### 3.4. Effects of PP on Body Weight, Serum Biochemistry, and Histology

These network pharmacology results suggested that PP may modulate multiple pathways related to lipid metabolism and inflammatory responses. To validate these predicted pharmacological effects, the protective effects of PP were further evaluated in an HFD-induced mouse model.

**Figure 3:**
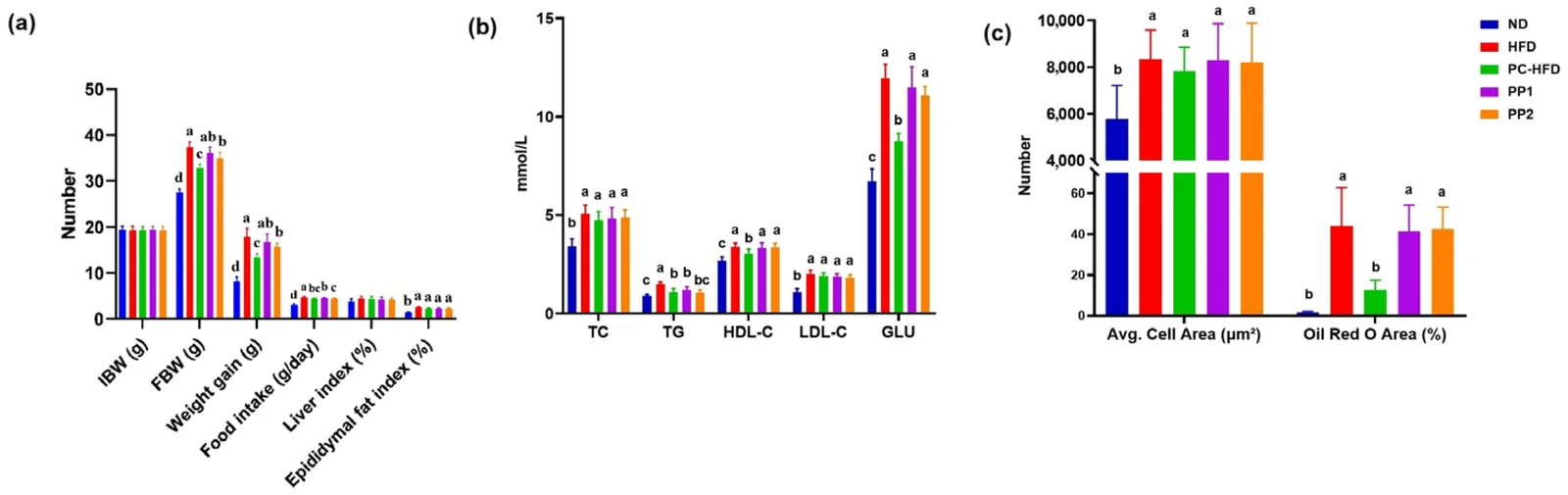
Effect of PP on metabolic phenotypes and tissue pathology in HFD-fed mice: (**a**) body weight and organ indices; (**b**) serum lipid profile and fasting blood glucose levels; and (**c**) mean adipocyte size in epididymal adipose tissue and the percentage area of hepatic lipid droplets (Oil Red O staining). Data are presented as mean ± SD (*n* = 6). Bars sharing different lowercase letters (a, b, c, and d) are statistically different (*p* < 0.05). IBW, initial body weight; FBW, final body weight; TC, total cholesterol; TG, triglyceride; HDL-C, high-density lipoprotein cholesterol; LDL-C, low-density lipoprotein cholesterol; GLU, blood glucose.

The results showed that PP intervention significantly suppressed excessive body weight gain induced by a high-fat diet, as evidenced by significantly reduced final body weight and body weight gain in PP-treated mice compared with the HFD group (Figure 3a). Although no statistically significant difference was observed between the two intervention groups (PP1 and PP2), the body weight gain in the PP2 group was numerically lower than that in the PP1 group. Regarding food intake, both PP1 and PP2 interventions significantly decreased the daily food intake compared with the HFD group. Analysis of glucose and lipid metabolism revealed that PP intervention significantly reduced serum triglyceride (TG) levels. However, no statistically significant difference was observed between the PP1 and PP2 groups. Meanwhile, changes in other serum lipid parameters and fasting blood glucose showed a trend toward improvement compared to the HFD group, but these changes did not reach statistical significance (Figure 3b). However, histopathological evaluation showed that PP intervention had no significant effect on the average adipocyte size in epididymal adipose tissue or on hepatic lipid deposition (measured as the percentage of Oil Red O-positive area) (Figure 3c and Figure S2). Serum liver-injury markers (ALT, AST, and ALP) were not measured in this study, limiting the comprehensive assessment of hepatic function and its correlation with histopathological findings. Future studies will prioritize these measurements to validate the hepatoprotective effects.

Compared with the HFD group, mice supplemented with PP exhibited significantly reduced body weight, body-weight gain, food intake, and serum TG levels. Our observed body-weight and TG reductions are consistent with the anti-dyslipidemic effects of *P. incarnata* leaf extracts reported by Sarto et al. (2018), who found that a dry extract (200 mg/kg/day, 30 days) lowered TC, TG, and C-reactive protein in HFD-fed LDLr^−^/^−^ mice [14]. These regulatory effects on body weight and lipid metabolism have been widely validated in other polyphenol intervention studies. For instance, blueberry polyphenols inhibit body weight gain and improve lipid metabolism in HFD-induced obese mice [25]. Longan byproduct polyphenol extracts reversed body weight gain by 11.8%, accompanied by comprehensive reductions in serum TC, TG, and LDL levels, as well as inhibition of hepatic lipid accumulation [26]. Additionally, lotus root soluble dietary fiber–polyphenol complexes not only effectively suppressed HFD-induced body-weight gain but also exhibited combined effects in regulating lipid profiles, particularly in reducing TG and increasing HDL [27]. Naringenin and naringin (20–100 µM) reduced TG and lipid accumulation in 3T3-L1 cells via phosphorylation of AMP-activated protein kinase (AMPK) and acetyl-CoA carboxylase [28].

### 3.5. Effects of PP on Serum Inflammatory Cytokines and Liver Antioxidant Enzymes Activity

**Figure 4:**
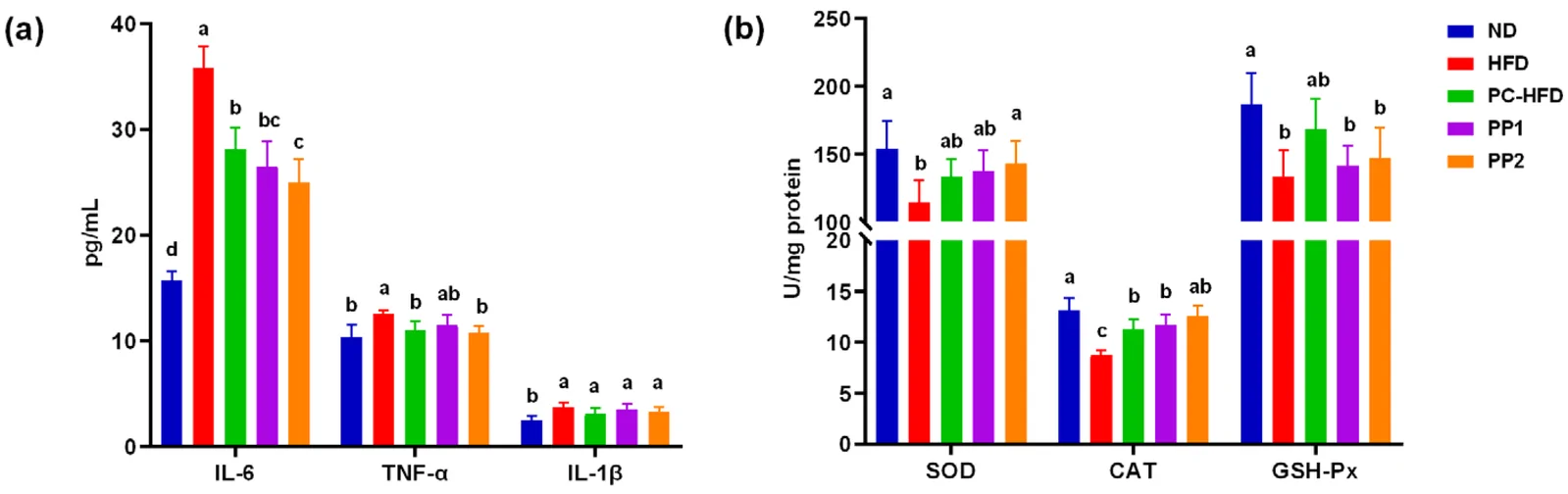
Effects of PP on systemic inflammation and hepatic antioxidant status in HFD-fed mice: (**a**) serum levels of interleukin-6 (IL-6), tumor necrosis factor-α (TNF-α), and interleukin-1β (IL-1β); and (**b**) hepatic activities of superoxide dismutase (SOD), catalase (CAT), and glutathione peroxidase (GSH-Px). Data are presented as mean ± SD (*n* = 6). Bars bearing different lowercase letters (a, b, c, and d) are statistically different (*p* < 0.05).

In terms of anti-inflammatory effects (Figure 4a), compared with the HFD group, PP intervention significantly reduced serum pro-inflammatory cytokine levels. The PP2 group showed a numerically greater reduction in IL-6 and TNF-α levels compared to the PP1 group, but the difference between the two PP groups was not statistically significant. Specifically, the PP2 group significantly decreased interleukin-6 (IL-6) levels by approximately 30.2% (*p* < 0.05) and tumor necrosis factor-alpha (TNF-α) levels by approximately 11.7%, while the PP1 group achieved reductions of 25.9% for IL-6 and 7.7% for TNF-α. However, PP intervention did not significantly reduce interleukin-1β (IL-1β) levels, which only showed a slight downward trend.

Regarding antioxidant effects (Figure 4b), PP intervention effectively reversed the HFD-induced suppression of the hepatic antioxidant enzyme system. Compared with the HFD group, both PP1 and PP2 significantly increased the activities of superoxide dismutase (SOD; by 20.5% and 25.0%, respectively) and catalase (CAT; by 34.0% and 43.9%, respectively). Additionally, both PP1 and PP2 enhanced glutathione peroxidase (GSH-Px) activity (by 33.8% and 30.5%, respectively); however, this increase did not reach statistical significance compared to the HFD group. Notably, the PP2 group exhibited superior or comparable efficacy to the positive control (PC) group in enhancing SOD and CAT activities, with all measured enzyme activities showing a tendency to recover toward the levels of the normal control (ND) group.

The present study demonstrates that PP intervention, particularly at the higher dose (PP2), significantly reduces serum levels of the pro-inflammatory cytokines IL-6 and TNF-α and enhances the activities of key hepatic antioxidant enzymes SOD and CAT, while exhibiting a non-significant upward trend in GSH-Px activity in HFD-fed mice. These findings indicate that PP exerts a clear beneficial effect in alleviating obesity-associated low-grade systemic inflammation and hepatic oxidative stress. This observation aligns with the well-documented effects of various dietary polyphenols. For instance, P. ligularis extract, resveratrol (400 mg/kg/day), and a quercetin–resveratrol combination have all been reported to suppress the production of inflammatory mediators such as TNF-α and IL-6 [29,30,31]. Similarly, tea polyphenols, quercetin, and sugarcane polyphenol extracts have been widely shown to enhance the activities of endogenous antioxidant enzymes, including SOD, CAT, and GSH-Px, often in a dose-responsive manner [32,33,34]. These common observations underscore the fundamental role of polyphenols in modulating inflammatory and oxidative balance.

Mechanistically, the anti-inflammatory and antioxidant effects of polyphenols are complementary and are often achieved through a dual mechanism involving both molecular and microbial pathways. At the molecular level, polyphenols can directly regulate key signaling pathways such as NF-κB and Nrf2, suppressing the expression of pro-inflammatory cytokines and activating endogenous antioxidant defense systems [35,36]. At the gut microbiota level, polyphenols exert their effects by enriching beneficial bacteria (e.g., Lactobacillus and Bifidobacterium), increasing short-chain fatty acid (e.g., butyrate) production, repairing the intestinal barrier, and reducing endotoxemia, thereby systemically alleviating inflammation and oxidative stress at the “upstream” level [37,38,39]. The observed reduction in systemic inflammation and enhancement of hepatic antioxidant capacity in this study may be associated with such multi-target and multi-level regulation.

### 3.6. PP Modulates the Gut Microbiota Composition in HFD-Fed Mice

**Figure 5:**
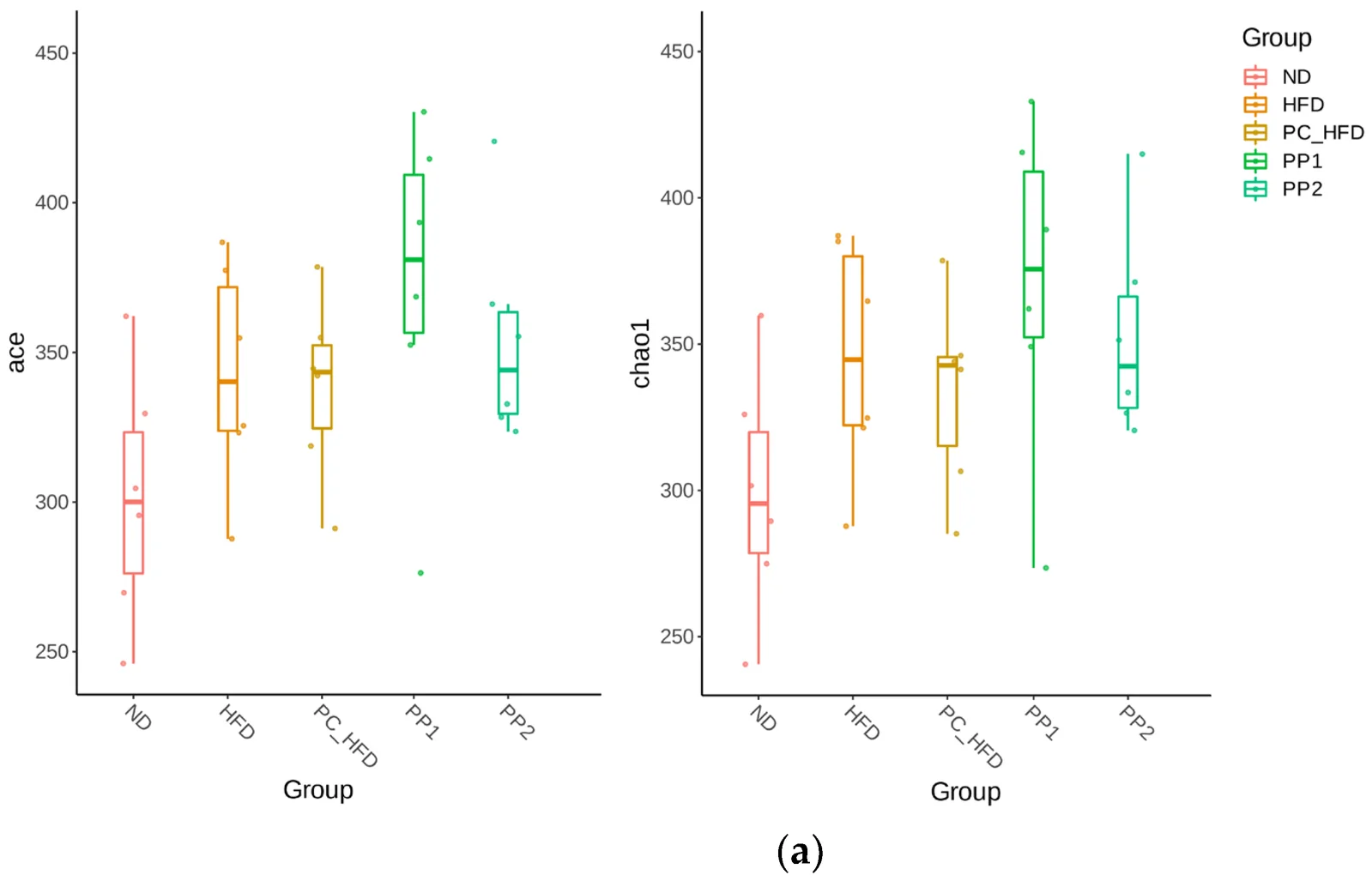

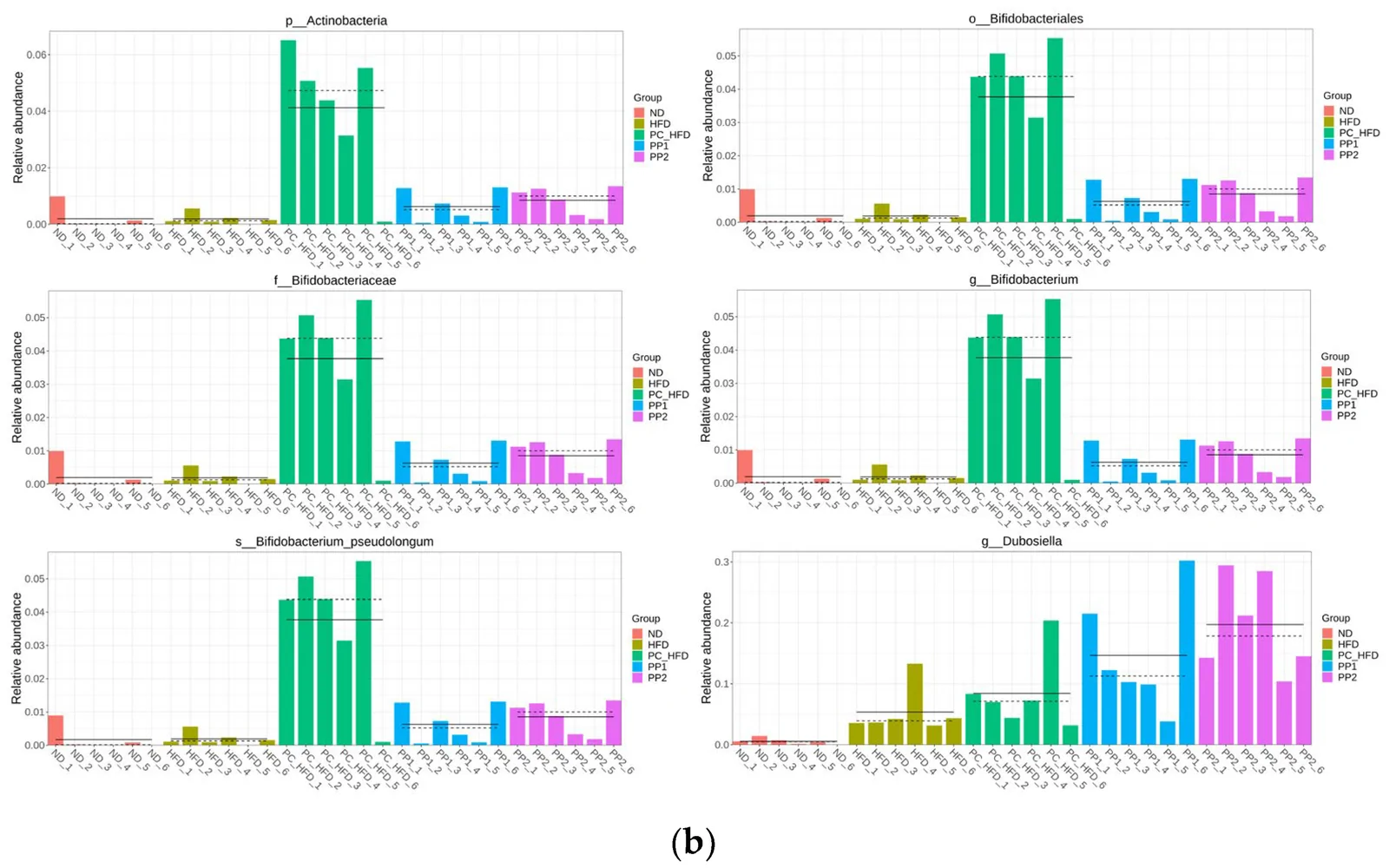
Gut microbiota analysis: (**a**) alpha-diversity indices (ACE and Chao1); and (**b**) LEfSe analysis revealing specific microbial abundance among different groups, (*n* = 6). The solid and dashed horizontal lines within each group indicate the mean and median relative abundance, respectively.

Among the five groups, both PP1 and PP2 supplementation displayed a non-significant upward trend in ACE and Chao1 indices compared with the HFD group, particularly notable in the PP1 group, which showed higher median diversity values (Figure 5a). Furthermore, 16S rRNA sequencing revealed a significant restructuring of the microbial community upon PP intervention (Figure 5b). Notably, there was a coordinated enrichment of a probiotic lineage within the phylum *Actinobacteria*, spanning the order *Bifidobacteriales*, family *Bifidobacteriaceae*, and genus *Bifidobacterium*, down to the species *Bifidobacterium pseudolongum*. In parallel, the abundance of *Dubosiella*, a genus associated with butyrate production, was also significantly elevated. The PP-induced enrichment of *Bifidobacterium* observed here aligns with conserved effects of plant polyphenol extracts on the gut microbiota. Green-tea polyphenols (0.05–0.2% of diet) increased *Bifidobacterium* and SCFA producers (acetate/butyrate) in HFD-fed human flora-associated (HFA) C57BL/6J mice [16]. Blueberry polyphenols enriched Bifidobacterium and elevated fecal SCFAs in HFD-fed C57BL/6J mice [25]. Grape-seed proanthocyanidins (200 mg/kg/day for 8 weeks) reshaped gut microbiota composition and elevated colonic acetate, propionate, and butyrate [17].

Gut microbiota dysbiosis is a core feature of obesity and its associated metabolic disorders, and fecal microbiota transplantation studies have directly established its causal role in disease pathogenesis [40]. In this study, we found that PP intervention specifically remodeled the gut microbiota structure in HFD-fed mice, inducing a multi-level, synergistic enrichment of the probiotic Bifidobacterium lineage. Concurrently, the abundance of Dubosiella, a genus closely associated with butyrate production, was also significantly increased. These two enriched bacterial groups exhibit complementary functions: Bifidobacterium is a well-recognized probiotic that produces acetate and lactate; it improves intestinal barrier function, and its abundance is directly correlated with metabolic improvement and inflammation alleviation [41,42]. Dubosiella is a key butyrate producer [43], and butyrate is essential for maintaining intestinal homeostasis and exerting systemic anti-inflammatory effects [44]. Therefore, the PP-induced gut microbiota remodeling, characterized by the enrichment of the Bifidobacterium lineage and butyrate-producing bacteria, provides a potential microbiological basis for the observed reduction in serum inflammatory markers.

Beyond a conventional prebiotic effect, the PP-induced enrichment of the complete Bifidobacterium lineage may be associated with gut–adipose crosstalk relevant to adipose thermogenesis, although a functional role has not been established. Recent studies have revealed that the gut microbiota is a key mediator in regulating adipose tissue “browning” and thermogenesis, constituting a “gut–adipose” axis [45,46]. Dietary polyphenols induce white adipose tissue browning and enhance UCP1-mediated thermogenesis by modulating the composition and activity of the gut microbiota [47,48]. Notably, specific probiotic strains can directly participate in this process. For instance, two Bifidobacterium strains (*B. bifidum* DS0908 and *B. longum* DS0950) have been shown to promote white adipocyte browning and inhibit lipid accumulation by activating the PKA/p38 MAPK signaling pathway in adipose tissue [49]. The co-occurrence enrichment observed in this study, spanning from order to species level, is consistent with the possibility that PP is associated with a more stable and potentially functionally relevant “pro-thermogenic” gut microbial ecology; however, this interpretation requires functional validation. Based on these findings, we propose a speculative hypothesis: the systemic enrichment of the Bifidobacterium lineage by PP may contribute to anti-inflammatory effects through its metabolites (e.g., SCFAs) and may be associated with gut–adipose communication related to the thermogenic program in adipose tissue. However, the present data do not establish that this enrichment acts as a microbial signaling mediator or represents a necessary microecological foundation for thermogenic activation.

### 3.7. PP Remodels Fecal Metabolome and Key Metabolites in HFD-Fed Mice

**Figure 6:**
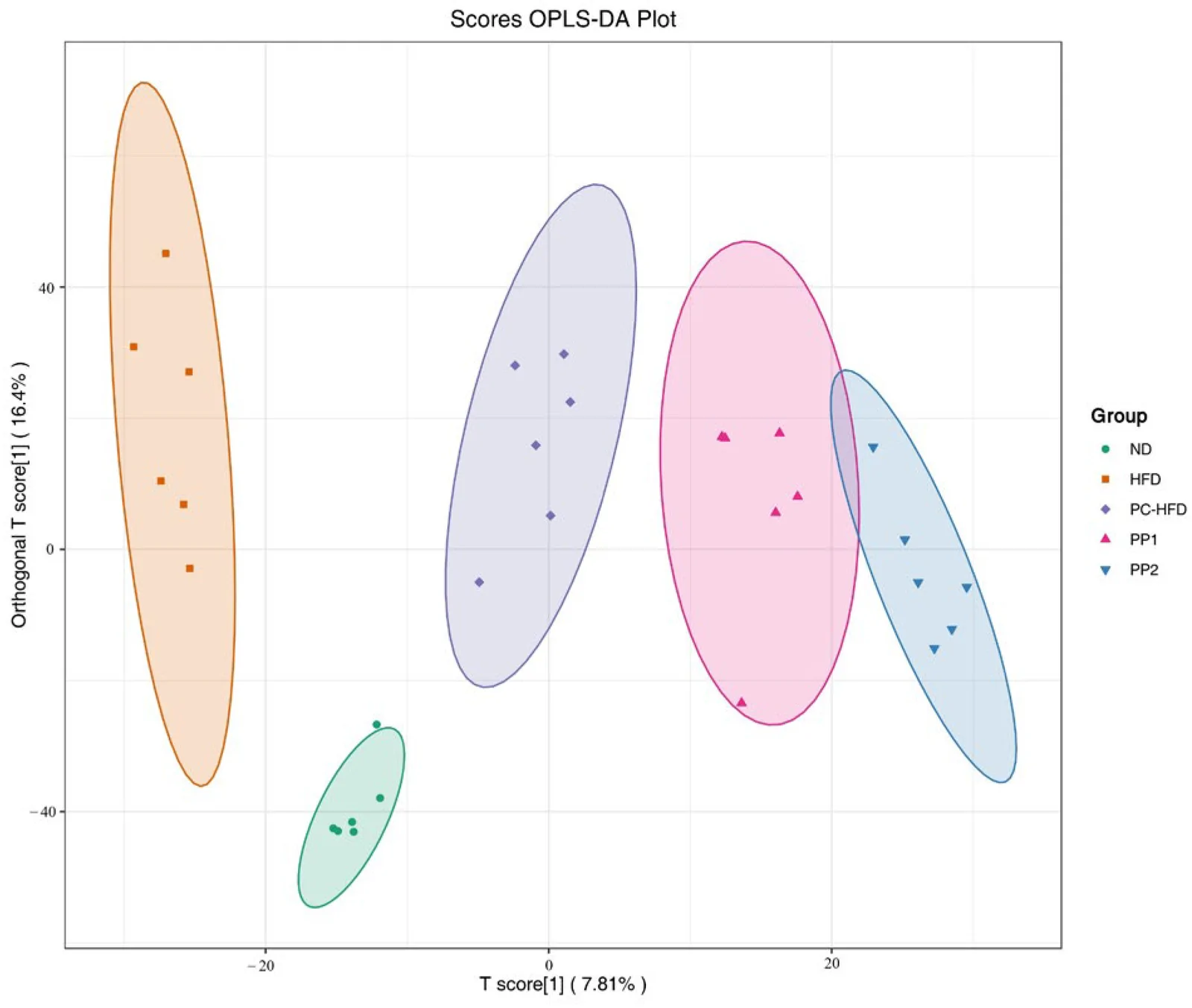
OPLS-DA analysis of fecal metabolomics in mice (*n* = 6).

Multivariate metabolic profiling using OPLS-DA demonstrated a distinct separation of fecal metabolic profiles among the experimental groups (Figure 6).

**Figure 7:**
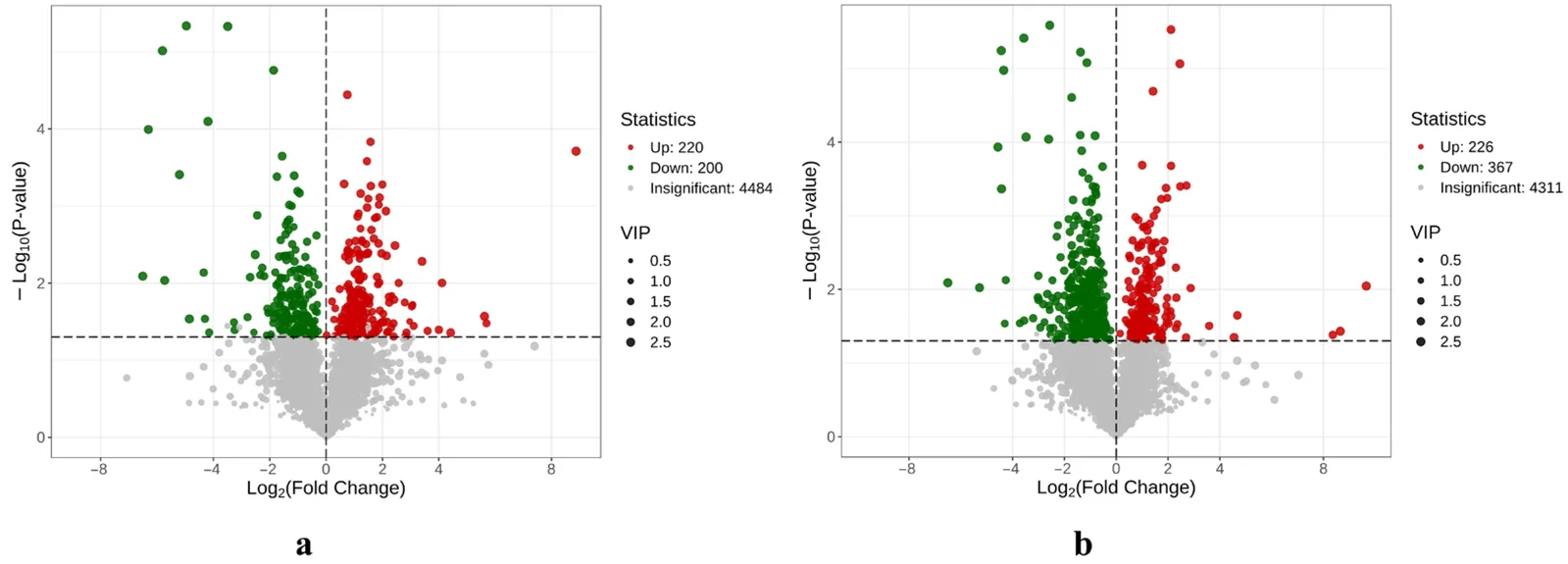
Volcano plot of the differential metabolites in mice: (**a**) PP1 vs. HFD and (**b**) PP2 vs. HFD.

Samples exhibited high intra-group aggregation and clear inter-group separation. Notably, the PP treatment groups formed distinct, non-overlapping clusters compared to the ND, HFD, and PC-HFD groups, indicating that PP intervention was associated with a marked alteration of the fecal metabolome. Volcano plot analysis further revealed the extent of this modulation: compared to the HFD group, PP1 intervention resulted in 220 upregulated and 200 downregulated metabolites, while PP2 intervention led to 226 upregulated and 367 downregulated metabolites (Figure 7). The larger number of downregulated metabolites in the PP2 group compared to PP1 suggests a possible dose-related effect on specific metabolic processes induced by the HFD.

**Figure 8:**
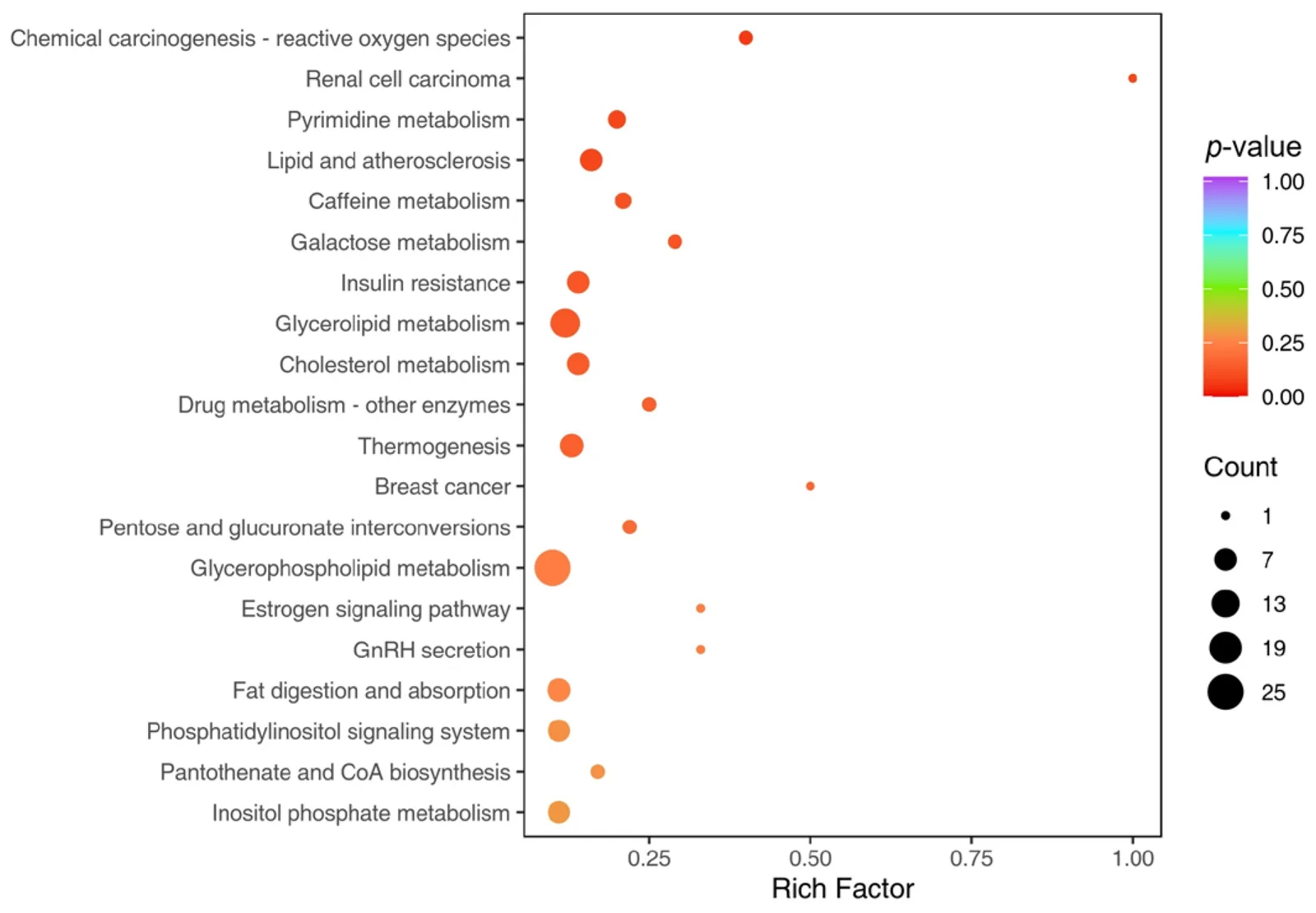
KEGG pathway enrichment analysis of differential metabolites in mouse feces across experimental groups (HFD vs. PC-HFD vs. PP1 vs. PP2). The bubble plot illustrates the top 20 most significantly enriched metabolic pathways.

**Figure 9:**
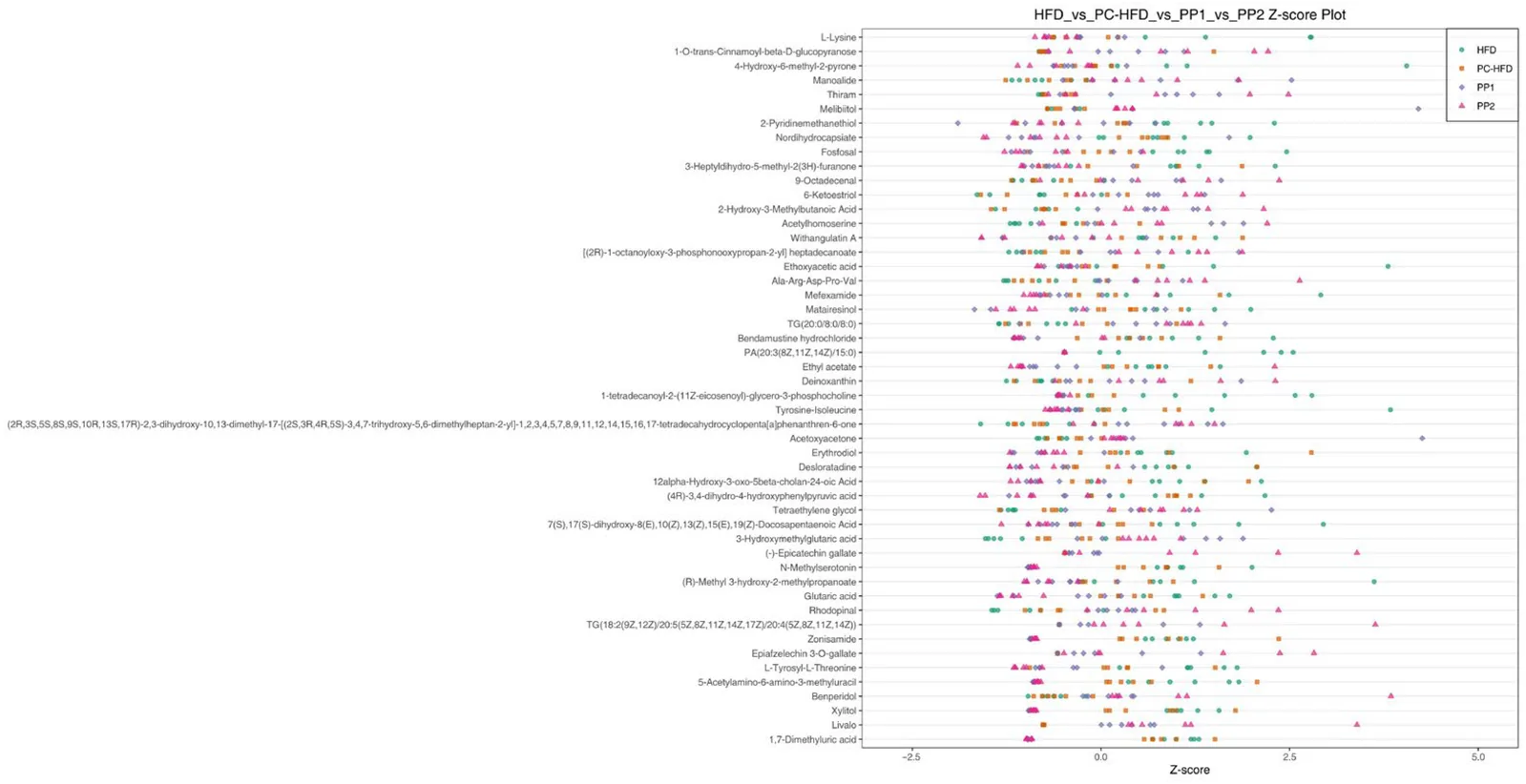
Z-score visualization of key differential metabolites in mouse feces across experimental groups (HFD vs. PC-HFD vs. PP1 vs. PP2).

Pathway enrichment analysis (KEGG) and Z-score visualization of key metabolites revealed enrichment of multiple pathways. We interpret these patterns as suggesting that PP may exert a multi-pathway regulatory effect and may contribute to metabolic homeostasis. (Figure 8 and Figure 9). Specifically, the significant enrichment of the “chemical carcinogenesis–reactive oxygen species” pathway represents a candidate mechanism that could underlie the observed enhancement of hepatic antioxidant capacity; however, this remains a correlative inference requiring direct experimental validation. This observation is consistent with the significantly increased activities of hepatic SOD and CAT observed in this study, although formal correlation analysis was not performed. Polyphenolic compounds are well-known potent antioxidants capable of upregulating the expression of endogenous antioxidant enzymes, including SOD and CAT, by activating the NRF2 signaling pathway, thereby systemically enhancing the body’s antioxidant defense capacity [50,51].

In terms of energy expenditure, “thermogenesis” pathway, centered on UCP1-mediated adaptive thermogenesis [52], was observed. Previous studies have indicated that specific polyphenols can enhance UCP1-dependent thermogenesis by activating signaling axes such as AMPK-PGC-1α, thereby promoting energy expenditure [53]. Thus, the enrichment of this pathway in the fecal metabolome raises the possibility that PP may influence systemic energy expenditure; however, this hypothesis requires further investigation.

Regarding lipid metabolism and burden reduction, PP intervention significantly altered lipid metabolism. The enrichment of “cholesterol metabolism” and “fat digestion and absorption” pathways, coupled with the increased fecal excretion of specific TGs (e.g., TG(18:2/20:5/20:4)), suggests a potential mechanism involving reduced lipid absorption [54]. Furthermore, PP intervention downregulated L-lysine (a marker of metabolic overload) and glutaric acid (a mitochondrial toxin), while upregulating beneficial bioactives such as (–)-epicatechin gallate (antioxidant) and manoalide (anti-inflammatory). This shift suggests a potential shift in amino acid flux and mitochondrial function, as previously proposed in related contexts [55,56,57,58]. However, these functional interpretations, while plausible, remain speculative and require direct experimental validation.

**Figure 10:**
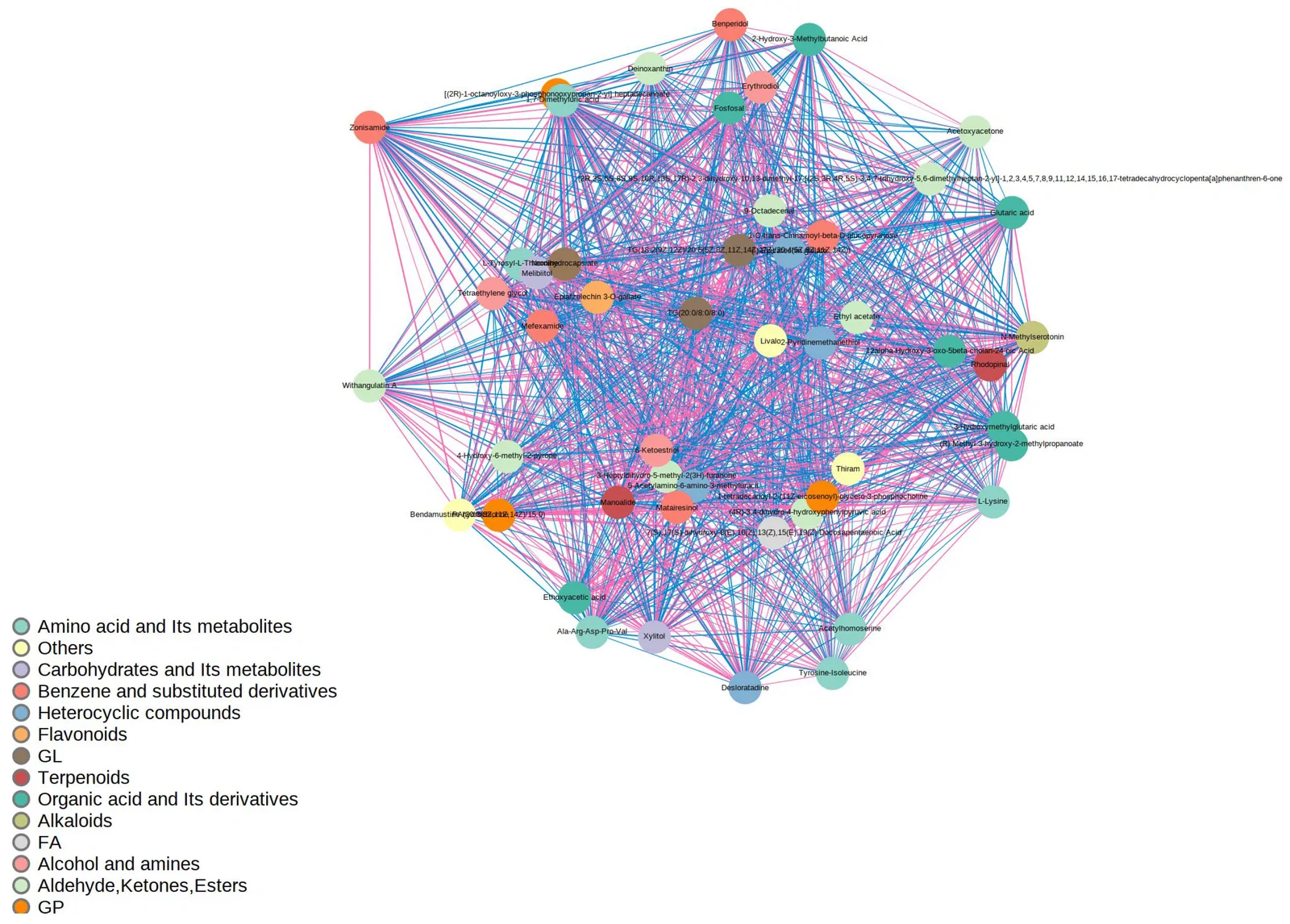
Pearson correlation network of differential metabolites. Nodes represent differential metabolites, colored by chemical classes. Edges represent statistically significant correlations (*p* < 0.05) between metabolites, with pink and blue edges indicating positive and negative correlations, respectively. GL, glycerolipid; FA, fatty acid; GP, glycerophospholipid.

**Figure 11:**
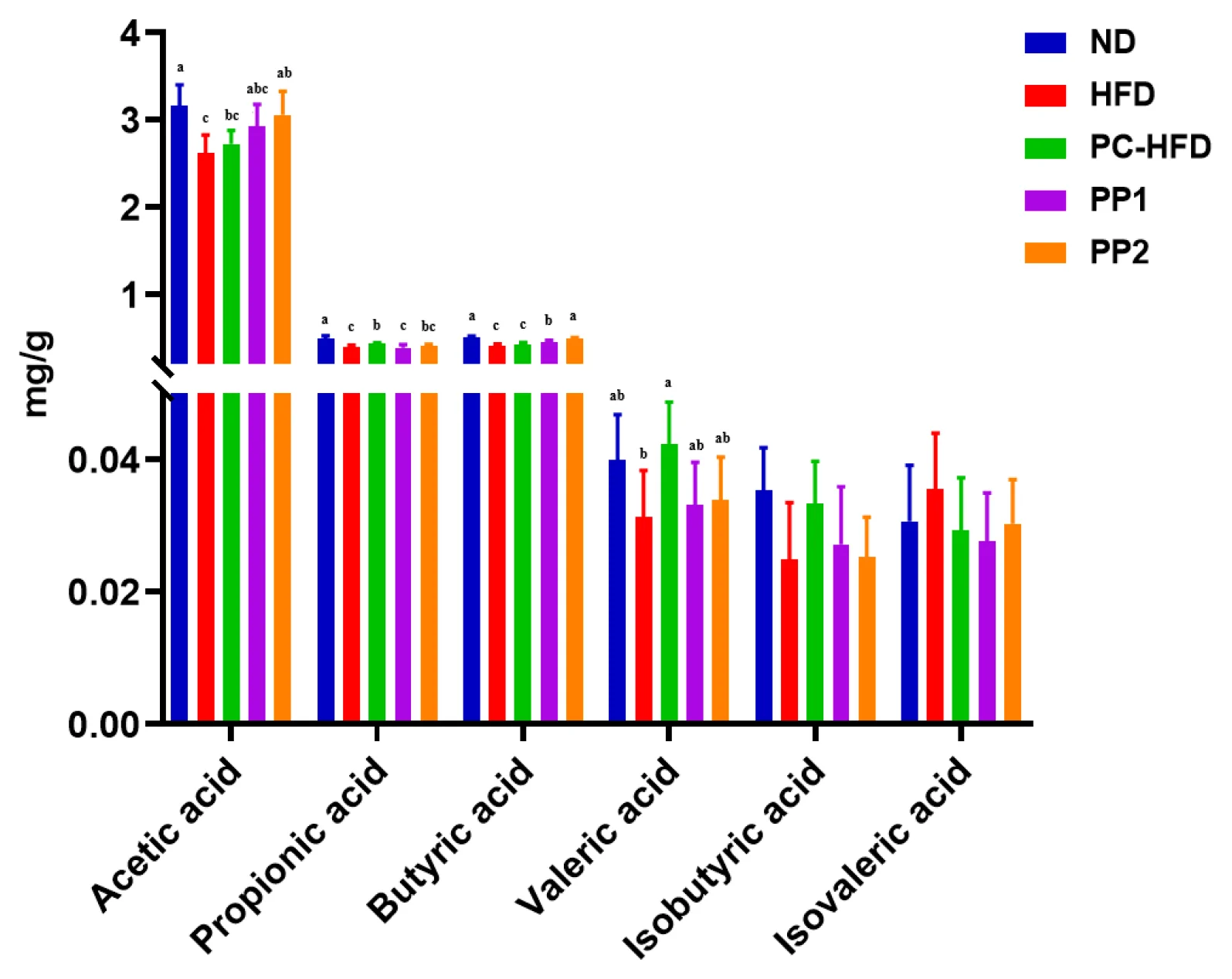
Effects of PP on fecal short-chain fatty acids profiles, *n* = 6. Bars with different superscript letters (a, b, c) indicate statistically significant differences among groups (*p* < 0.05).

Correlation network analysis and SCFA quantification further support a systemic, network-level regulation involving host–microbe interactions (Figure 10 and Figure 11). The correlation network revealed extensive cross-class connectivity among differential metabolites, indicating that PP’s impact is systemic rather than pathway-specific. Functionally, PP intervention significantly increased fecal levels of acetic acid and butyric acid, restoring them to levels close to the ND group. This metabolic shift correlates with the 16S rRNA data showing enrichment of Bifidobacterium and Dubosiella, genera known for producing these SCFAs. This association provides a correlative link between PP-induced microbiota remodeling and the observed metabolic improvements, though causality cannot be inferred from these data alone. In the metabolomic analysis, although the OPLS-DA models were validated by R^2^X, R^2^Y, Q^2^ (Q^2^ > 0.5), and a 200-fold permutation test (*p* < 0.05), differential metabolites were selected at nominal *p* < 0.05 (VIP > 1) without additional multiple-comparison correction; therefore, these metabolomic findings should be regarded as exploratory.

### 3.8. PP Ameliorates Hepatic Metabolic Disorders with Dose-Associated Transcriptomic Reprogramming in HFD-Fed Mice

**Figure 12:**
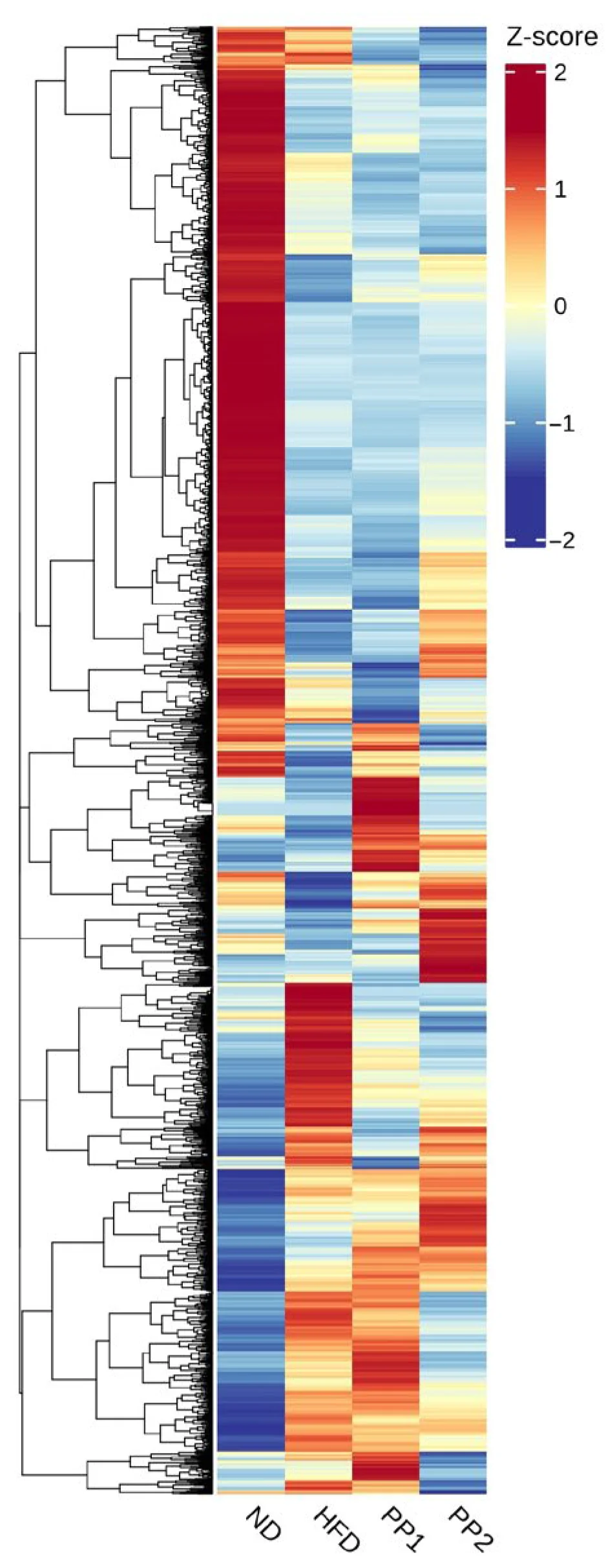
Hierarchical clustering heatmap of differentially expressed genes (DEGs) in liver tissue, *n* = 6.

Global transcriptomic profiling revealed a clear divergence in hepatic gene expression patterns among the ND, HFD, and PP-treated groups (Figure 12). Hierarchical clustering analysis demonstrated a distinct separation between the HFD group and the ND group, indicating that the high-fat diet induced substantial disruptions in the liver transcriptome. Compared to the ND baseline, the HFD group was characterized by extensive transcriptional dysregulation, involving both profound downregulation (deep blue) and obvious upregulation (deep red) across distinct gene clusters, rather than merely broad transcriptional suppression. In contrast, both PP1 and PP2 interventions shifted the expression profiles away from the HFD pattern. Z-score visualization showed that PP treatment counteracted the HFD-induced perturbations through a dual regulatory pattern: it reversed the HFD-suppressed clusters toward upregulation, while simultaneously attenuating the HFD-overexpressed clusters back toward the ND baseline.

**Figure 13:**
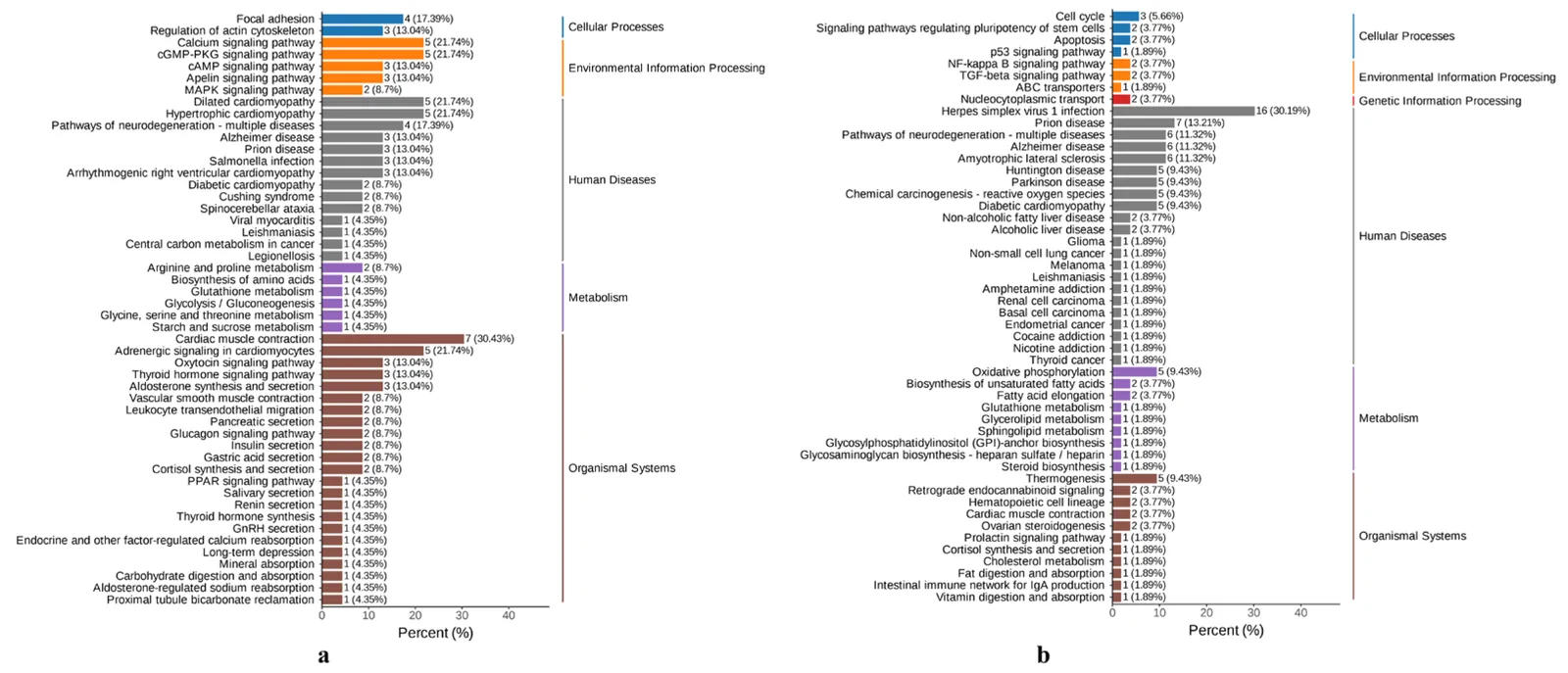
KEGG pathway enrichment analysis of differentially expressed genes in the liver of PP1 and PP2 groups compared with HFD group. (**a**) PP1 vs. HFD and (**b**) PP2 vs. HFD.

Transcriptomic analysis further suggested a dose-associated regulatory effect of PP on hepatic metabolic pathways (Figure S1 and Figure 13). For the liver transcriptome, differentially expressed genes were defined using the pre-specified threshold of |log_2_Fold Change| ≥1 and Benjamini–Hochberg-adjusted FDR < 0.05, providing stringent false-positive control. Compared to the HFD group, PP1 intervention identified 60 differentially expressed genes (DEGs), comprising 54 upregulated and six downregulated genes. In contrast, the high-dose PP2 intervention induced a significantly broader response, identifying 138 DEGs (111 upregulated and 27 downregulated), approximately 2.3 times the number observed in the PP1 group. This quantitative difference between the two doses suggests that higher PP dosage may be required to trigger more extensive transcriptional changes, though formal dose–response testing was not performed.

Functional enrichment analysis of these DEGs delineated distinct regulatory layers corresponding to the dosage. In the high-dose PP2 group, DEGs were significantly enriched in three major functional clusters, thereby confirming the metabolic improvements observed phenotypically. Specifically, enrichment of the “chemical carcinogenesis–reactive oxygen species” and “glutathione metabolism” pathways corroborated the increased hepatic activities of SOD and CAT, supporting the notion that PP2 may mitigate oxidative stress at the transcriptional level. Concurrently, the enrichment of both “oxidative phosphorylation” and “thermogenesis” pathways raises the possibility that PP2 may be associated with mitochondrial energy synthesis and energy expenditure programs, which could contribute to the observed hepatic metabolic improvements, although pathway enrichment alone does not establish functional activation. Furthermore, the broad modulation of pathways associated with cellular stress and inflammation may reflect a concurrent alleviation of the pathological states induced by HFD.

In contrast, the PP1 group exhibited a distinct enrichment pattern, primarily involving fundamental cellular maintenance pathways. DEGs in the PP1 group were mainly associated with cytoskeletal regulation (13.04%), focal adhesion (17.39%), and cardiac muscle contraction (30.43%). These pathways are typically linked to basic cell morphology and cell–matrix interactions. Unlike PP2, PP1 did not show significant enrichment in core metabolic pathways such as oxidative phosphorylation or thermogenesis. This differential response pattern suggests that while low-dose PP1 may induce basic cellular adaptation, a higher dose (PP2) may be necessary to elicit more pronounced metabolic reprogramming, a pattern commonly observed in natural product pharmacology [59].

### 3.9. PP Modulates Thermogenic and Regulatory Gene Expression in Adipose Tissue of HFD-Fed Mice

**Figure 14:**
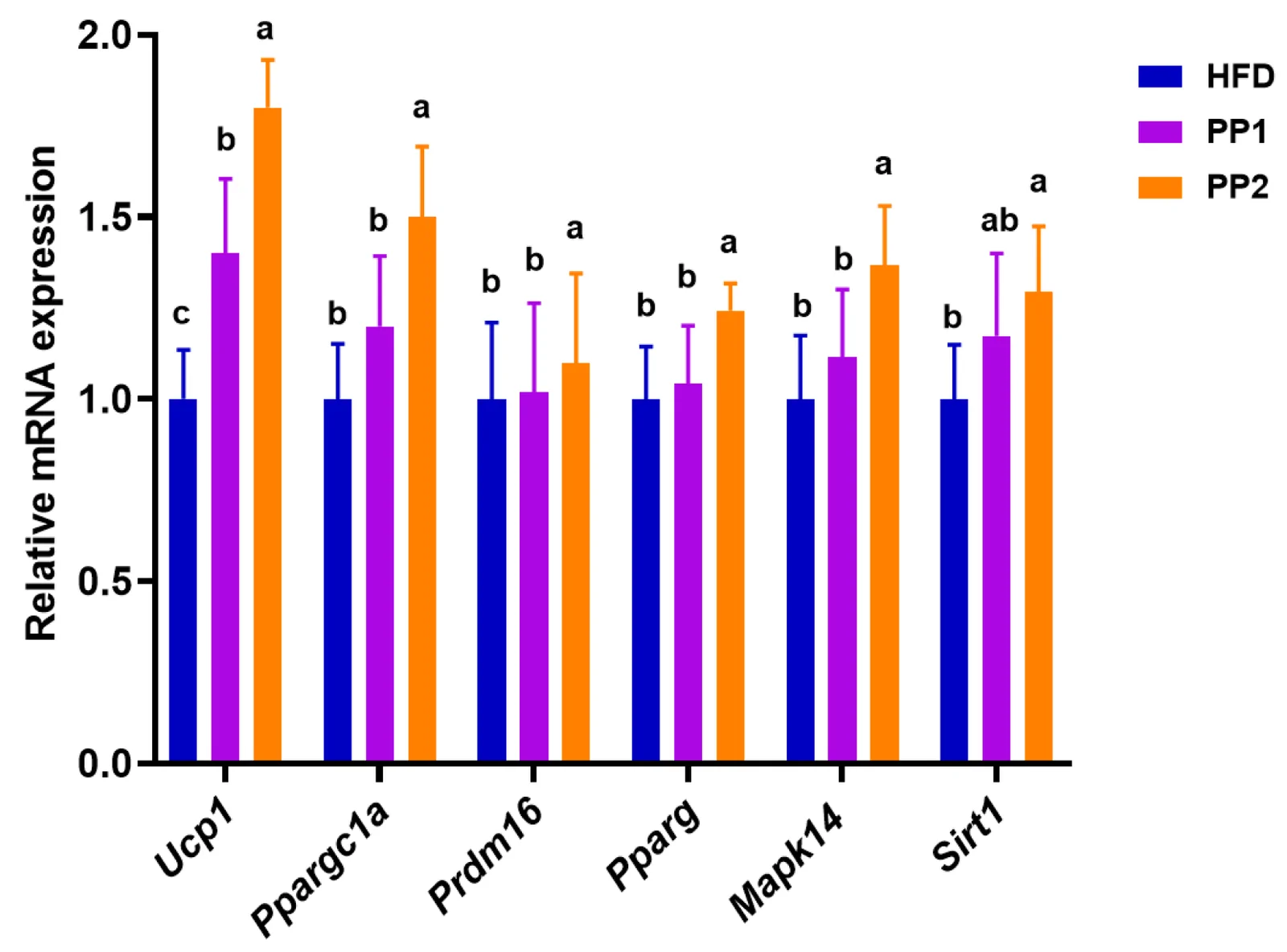
Relative mRNA expression levels of key thermogenic and regulatory genes in the adipose tissue of mice from different treatment groups. The genes analyzed were *Ucp1*(uncoupling protein 1), *Ppargc1a* (PPARγ coactivator 1-alpha), *Prdm16* (PR domain containing 16), *Pparg* (peroxisome proliferator-activated receptor gamma), *Mapk14* (mitogen-activated protein kinase 14)/p38α, and *Sirt1* (sirtuin 1), *n* = 6. Bars with different superscript letters (a, b, c) indicate statistically significant differences among groups (*p* < 0.05).

Based on the observed associations between the enriched *Bifidobacterium* lineage and host metabolic parameters, together with the enrichment of thermogenesis-related pathways in fecal metabolomics and hepatic transcriptomics, we further investigated whether PP could be associated with changes in the thermogenic gene program in adipose tissue. Of note, the ND group was not included in this analysis; therefore, the results reflect the modulatory effects of PP relative to the HFD group rather than a restoration to a healthy baseline. As shown in Figure 14, qRT-PCR analysis revealed a dose-associated effect of passion fruit leaf polyphenols on the regulation of thermogenic gene expression in adipose tissue. High-dose intervention (PP2) broadly and significantly upregulated the mRNA expression of a series of key genes, including the core thermogenic effector protein (*Ucp1*), the master regulator of mitochondrial biogenesis (*Ppargc1a*), the browning determinant (*Prdm16*), the key adipogenic regulator (*Pparg*), the stress signaling kinase (*Mapk14*), and the metabolic sensor (*Sirt1*). Among these, the induction of Ucp1 and Ppargc1a was particularly prominent. In contrast, the effect of low-dose intervention (PP1) was more limited, significantly upregulating only Ucp1 expression (*p* < 0.05), with no significant effects on the mRNA levels of Ppargc1a, Prdm16, Pparg, Mapk14, or Sirt1.

**Figure 15:**
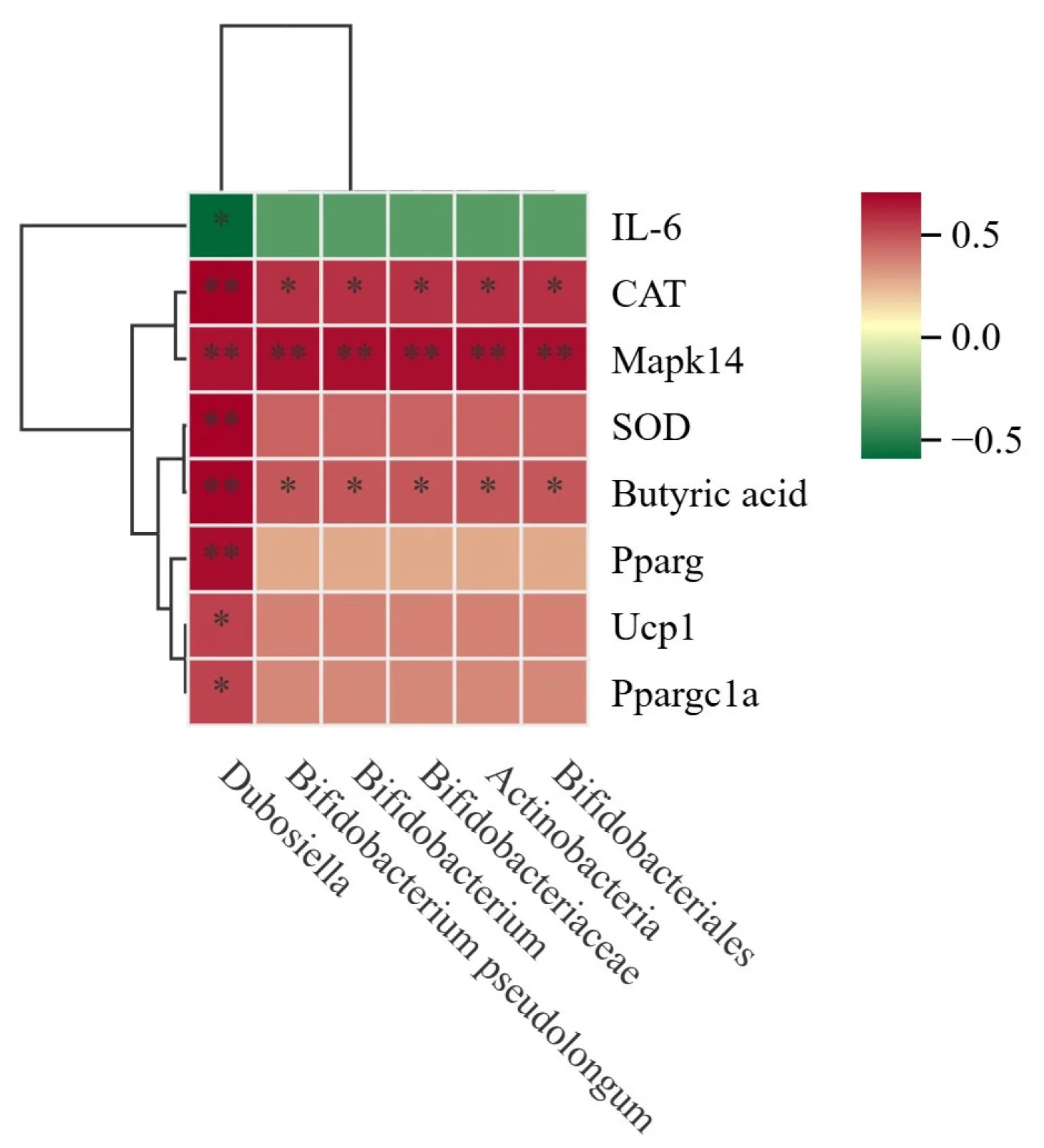
Spearman correlation heatmap between gut microbial taxa and host metabolic parameters. IL-6, interleukin-6; CAT, catalase; *Mapk14*, mitogen-activated protein kinase 14; SOD, superoxide dismutase; *Pparg*, peroxisome proliferator-activated receptor gamma; *Ucp1*, uncoupling protein 1; *Ppargc1a*, peroxisome proliferator-activated receptor gamma, coactivator 1 alpha. * *p* < 0.05, ** *p* < 0.01.

To determine whether these thermogenic changes were linked to the previously observed gut microbiota remodeling, we performed Spearman correlation analysis between key bacterial taxa and host metabolic parameters (Figure 15). The PP-enriched Bifidobacterium lineage (from order to species level) and Dubosiella exhibited robust positive correlations with fecal butyrate, hepatic antioxidants (CAT), and adipose thermogenic markers (*Ucp1*, *Ppargc1a*, *Pparg*, and *Mapk14*). In contrast, these beneficial taxa were negatively correlated with the pro-inflammatory cytokine IL-6. It is important to note that these correlation analyses do not establish causality; they only indicate statistical associations. Therefore, the following interpretations should be considered as hypotheses requiring future functional validation (e.g., via microbiota transplantation, SCFA supplementation, or gene knockout experiments).

With this caveat in mind, these results raise the possibility that PP2 is associated with a coordinated transcriptional pattern that is consistent with adipose tissue browning. Within this pattern, the deacetylase *SIRT1* is one candidate upstream regulator; however, the present data do not establish a central or initiating role for *SIRT1*. *SIRT1* has been reported to activate *PPARγ* and its coactivator *PGC-1α* through deacetylation, and this complex serves as a key transcriptional hub driving the thermogenic gene program, including *Ucp1* [60,61]. The coordinated upregulation of *Sirt1*, *Pparg*, *Ppargc1a*, and *Ucp1* in the PP2 group is consistent with this classical pathway, but mRNA co-expression alone cannot demonstrate upstream causality. *PGC-1α* is not only a key coactivator for *UCP1* expression [62]; it is also regulated by *SIRT1* [63], consistent with, but not proof of, an upstream regulatory role for SIRT1 in our experimental model. Moreover, the significant induction of Prdm16 by PP2 is consistent with findings from other polyphenol studies (e.g., quercetin) [64], suggesting that PP may be associated with a browning-related transcriptional program involving the *PPARγ*/*PRDM16* complex, although direct functional evidence is lacking.

The PP2-mediated coordinated upregulation of *Ucp1*, *Ppargc1a*, *Prdm16*, *Pparg*, and *Sirt1* in adipose tissue is consistent with conserved thermogenic effects of diverse plant polyphenols. Grape-seed proanthocyanidins (200 mg/kg/day, 8 weeks) elevated *UCP1* in BAT and *UCP1*/*PGC-1α*/*PRDM16* in iWAT in HFD C57BL/6J mice [17]. Apple polyphenols induced iWAT browning with *UCP1*/*CIDEA*/*TBX1* and *PGC-1α* upregulation [1]. Resveratrol activated the same axis via *AMPK*/*SIRT1*-dependent deacetylation of *PPARγ*, driving *PRDM16–PGC-1α–UCP1* expression [65]. These convergent observations across botanically distinct polyphenols suggest that the *SIRT1*-centered thermogenic axis may represent a common target for metabolic intervention.

Collectively, the positive correlations between butyrate-producing microbes and thermogenic genes (Figure 15) represent statistical associations that are consistent with, but do not establish, a possible link between PP-modulated gut microbiota, SCFA signaling, and the SIRT1-associated network. Together with the observed hepatic benefits, these correlative findings raise the possibility of a gut–liver–adipose association, in which changes in microbiota-derived SCFAs are statistically associated with hepatic antioxidant defense and adipose thermogenesis. However, it should be emphasized that the current evidence is based on correlation analyses and gene expression patterns; direct causal relationships between specific microbes, SCFAs, and the SIRT1 pathway have not been experimentally established in this study. Overall, our data indicate that PP, especially at the high dose, upregulates a thermogenic transcriptional network that includes SIRT1 in adipose tissue, and is compatible with, but does not confirm, a proposed gut–liver–adipose crosstalk framework based on these observed associations, pending future mechanistic validation.

## 4. Conclusions

PP supplementation showed dose-related trends in ameliorating HFD-induced metabolic disturbances, with PP2 tending to produce broader molecular changes than PP1, although formal dose–response relationships were not established. Integration of 16S rRNA sequencing, fecal metabolomics, liver transcriptomics, and adipose qPCR supports a correlative gut–liver–adipose crosstalk hypothesis: PP intervention was associated with decreased caloric intake, altered gut microbiota and fecal metabolome, enhanced hepatic antioxidant capacity, and enriched energy metabolism pathways, while PP2 was associated with upregulation of *SIRT1*/*UCP1*-related thermogenic genes in adipose tissue. The originality of this work lies in this multi-omics perspective, which captures the multi-target actions of *P*. *incarnata* polyphenols; however, possible synergy and causal relationships remain to be established by future interventional studies.

## Figures and Tables

**Table 1:** Primers used for quantitative real-time PCR.

Gene	Forward Primer	Reverse Primer
*Ucp1*	ACTGCCACACCTCCAGTCATT	CTTTGCCTCACTCAGGATTGG
*Ppargc1a*	CCGTAAATCTGCGGGATGATG	CAGTTTCGTTCGACCTGCGTAA
*Prdm16*	CACGTCTACGGTGAACGGAA	ATGGGATCCATGAAGAACGGT
*Mapk14*	ACACTCGGCTGACATAATCCA	CACGGACCAAATATCCACTGTCT
*Pparg*	GTCACACTCTGACAGGAGCC	CACCGCTTCTTTCAAATCTTGT
*Sirt1*	CTGGGGTTTCTGTCTCCTGT	AGGCGAGCATAGATACCGTC
*Gapdh*	GAAGGTCGGTGTGAACGGATTTG	CATGTAGACCATGTAGTTGAGGTCA

## Data Availability

The original contributions presented in this study are included in the article/Supplementary Materials. Further inquiries can be directed to the corresponding authors. The raw transcriptomic sequencing data generated in this study have been deposited into the Genome Sequence Archive (GSA) at the National Genomics Data Center (NGDC) under accession number CRA048824.

## Supplementary Materials

The following supporting information can be downloaded at https://www.mdpi.com/article/10.3390/foods15173078/s1, Supplementary Figure S1. Volcano plot of differentially expressed genes in mouse liver: (a) PP1 vs. HFD and (b) PP2 vs. HFD; Supplementary Figure S2. Representative histological images: (a) epididymal adipose tissue and (b) liver tissue; Table S1: Polyphenolic compounds detected in PP.
